# Classification and analysis of a large collection of *in vivo* bioassay descriptions

**DOI:** 10.1371/journal.pcbi.1005641

**Published:** 2017-07-05

**Authors:** Magdalena Zwierzyna, John P. Overington

**Affiliations:** 1 BenevolentAI, London, United Kingdom; 2 Institute of Cardiovascular Science, University College London, London, United Kingdom; Weizmann Institute of Science, ISRAEL

## Abstract

Testing potential drug treatments in animal disease models is a decisive step of all preclinical drug discovery programs. Yet, despite the importance of such experiments for translational medicine, there have been relatively few efforts to comprehensively and consistently analyze the data produced by *in vivo* bioassays. This is partly due to their complexity and lack of accepted reporting standards—publicly available animal screening data are only accessible in unstructured free-text format, which hinders computational analysis. In this study, we use text mining to extract information from the descriptions of over 100,000 drug screening-related assays in rats and mice. We retrieve our dataset from ChEMBL—an open-source literature-based database focused on preclinical drug discovery. We show that *in vivo* assay descriptions can be effectively mined for relevant information, including experimental factors that might influence the outcome and reproducibility of animal research: genetic strains, experimental treatments, and phenotypic readouts used in the experiments. We further systematize extracted information using unsupervised language model (Word2Vec), which learns semantic similarities between terms and phrases, allowing identification of related animal models and classification of entire assay descriptions. In addition, we show that random forest models trained on features generated by Word2Vec can predict the class of drugs tested in different *in vivo* assays with high accuracy. Finally, we combine information mined from text with curated annotations stored in ChEMBL to investigate the patterns of usage of different animal models across a range of experiments, drug classes, and disease areas.

## Author summary

Before exposing human populations to potential drug treatments, novel compounds are tested in living non-human animals—arguably the most physiologically relevant model system known to drug discovery. Yet, high failure rates for new therapies in the clinic demonstrate a growing need for better understanding of the relevance and role of animal model research. Here, we systematically analyze a large collection of *in vivo* assay descriptions—summaries of drug screening experiments on rats and mice derived from scientific literature of more than four decades. We use text mining techniques to identify the mentions of genetic and experimental disease models, and relate them to therapeutic drugs and disease indications, gaining insights into trends in animal model use in preclinical drug discovery. Our results show that text mining and machine learning have a potential to significantly contribute to the ongoing debate on the interpretation and reproducibility of animal model research through enabling access, integration, and large-scale analysis of *in vivo* drug screening data.

## Introduction

Testing potential therapeutic compounds in animal disease and safety models is a crucial part of preclinical drug discovery [1]. Although many *in vitro* methods have been developed to rapidly screen candidate molecules, no such simple assay system can recapitulate the complexities and dynamics of a living organism [2]. By contrast, an *in vivo* assay, depending on the animal species, allows a potentially far more realistic and predictive measure of a compound’s effect, and can capture the complexity of target engagement, metabolism, and pharmacokinetics required in the final therapeutic drug. Testing novel therapeutics *in vivo* is therefore most likely to accurately predict patient responses and successfully translate from bench to bedside [3]. In fact, a proof of efficacy and safety in animals is usually an essential requirement by regulatory agencies before progressing a compound into human studies [1, 4].

Drug efficacy tests are carried in animal models that mimic some aspects of human pathology. Based on how the disease state is created, animal models can be generally classified into three main groups [5]:

- In *experimental (induced) disease models*, the phenotype of interest is artificially induced with an experimental intervention such as electric shock, surgical procedure, behavioral training, or administration of specific chemicals [5]. For instance, mice injected with 1-methyl-4-phenyl-1,2,3,6-tetrahydropyridine (MPTP) rapidly develop pathologies reminiscent of Parkinson’s disease, as the chemical destroys their dopamine-producing neurons leading to uncontrollable tremors, bradykinesia, and other motor and behavioral deficits [6].
- *Genetic (or spontaneous) disease models* are genetic strains of animals that are primed to develop disease-related phenotypes due to some type of naturally occurring genetic variation. As an example, Lep^ob/ob^ mice develop hyperglycemia without any experimental intervention, as they become morbidly obese due to a point mutation in the gene for leptin—a hormone involved in regulation of energy use and inhibition of hunger [7].
- In *transgenic animal models*, mutations of disease-related genes are directly introduced with genome engineering techniques. This category includes many mouse knockout and knockdown models and is likely to gain further popularity due to technological developments, such as CRISPR/Cas9 genome editing methods [8].

Regardless of the type of an animal model used, the main purpose of *in vivo* drug screening is to offer useful insights into human biology and to predict human responses to novel treatments [9]. High attrition rates in the clinic, however, show that animal studies do not always reliably inform clinical research for both drug efficacy and safety [10]. Many scientists have drawn attention to the need for more systematic, rigorous, and objective analysis of animal research data before designing studies in patients [11, 12]. In particular, the probability of successful model species to human translation should be assessed based on careful meta-analyses and systematic reviews that integrate results across all relevant animal studies [9]. These should involve experiments performed on different strains, species, and experimental models [9], since they might recapitulate distinct aspects of human disease and offer variable accuracy of predictions. Several such systematic reviews performed retrospectively exposed various challenges of successful translation including publication bias, design flaws, insufficient reporting of experimental details, and lack of coordination between scientists involved in animal research and those designing clinical trials [13–15].

Currently, systematic reviews are performed manually and involve analysis of large quantities of published articles and internal proprietary reports. Attempts to automate some aspects of this time-consuming process have mainly focused on systematic identification and ranking of potentially relevant articles with robust search filters [16, 17] and machine learning methods [18]. More recently, Flórez-Vargas *et al*. used text mining to analyze the full text of 15,000 research papers describing mouse studies across a diverse range of therapeutic areas [19]. In addition to exposing insufficient reporting of gender and age of laboratory mice, the study found evidence of sex bias across specific fields of research. These results demonstrate the ability of text mining to offer insights into large-scale emergent trends and weaknesses of animal research by systematically analyzing large unstructured datasets [19, 20]. One discussed limitation of the analysis was due to the fact that details of animal experiments are typically reported in the full text of articles (as opposed to abstracts), which can only be obtained for open access publications [19], ~24% of biomedical research literature [21].

Our own study aims to contribute to the ongoing debate on the need for the integration of animal research data, not least in enabling discovery and reuse of previous research. As a basis of our analysis of *in vivo* drug testing information, we use ChEMBL [22]–a drug discovery focused bioactivity database widely known for its large curated and consistently indexed *in vitro* bioassay datasets. Animal model data in ChEMBL include descriptions and results of more than 100,000 drug screening experiments in rats and mice—the most widely used animal model species. These have been manually extracted by database curators from the full text of scientific articles.

In contrast to the molecular target annotated *in vitro* content of ChEMBL, its *in vivo* screening data are currently understudied and arguably under-curated. This is very likely due to their relative complexity compared to *in vitro* bioassays, and their inherent abstracted, unstructured format. The *in vivo* information is encoded in textual assay descriptions, written by database curators and intended for expert human users, not for computational analysis. The descriptions take the form of compact summary accounts such as: “*Inhibition of carrageenan-induced paw oedema in Sprague-Dawley rat at 5*.*16 mg/kg*, *sc after 3 hrs*”. In less than twenty words, the example above summarizes important details of the screening system (strain of the animal, experimental stimulus, phenotypic readout) as well as compound administration details (dosage, timing, and administration route). Hence—despite their concise format, assay descriptions can be information rich and we consider they have future potential in translational drug discovery research. However, the variety of possible expressions used to describe the same assay makes comparison and clustering of *in vivo* screening data from resources such as ChEMBL extremely challenging.

In this paper, we present the first, to our knowledge, computational analysis of the entire ChEMBL *in vivo* assay description dataset in rat and mouse. We use natural language processing (NLP) methods to parse the descriptions of *in vivo* assays and then mine them for information connecting animal models to human disease, genetic strains, experimental treatments, and phenotypes. To this end, we apply an approach that leverages existing community-maintained and stable vocabularies alongside manually crafted extraction rules. To automatically organize the extracted information, we construct a “semantic space” of assay descriptions using a neural network language model, and build several random forest (RF) classifiers. Finally, we show that combining information mined from text with structured curated data offers new and useful insights into the *in vivo* dataset in ChEMBL as well as trends in the use of animal models in drug discovery research in general. We restrict our current analysis to animal models used in the evaluation of the efficacy of drugs, not animal model usage in ADME or toxicology studies—although a similar analysis strategy can be applied to these.

## Results

### The ChEMBL *in vivo* dataset

**Fig 1:**
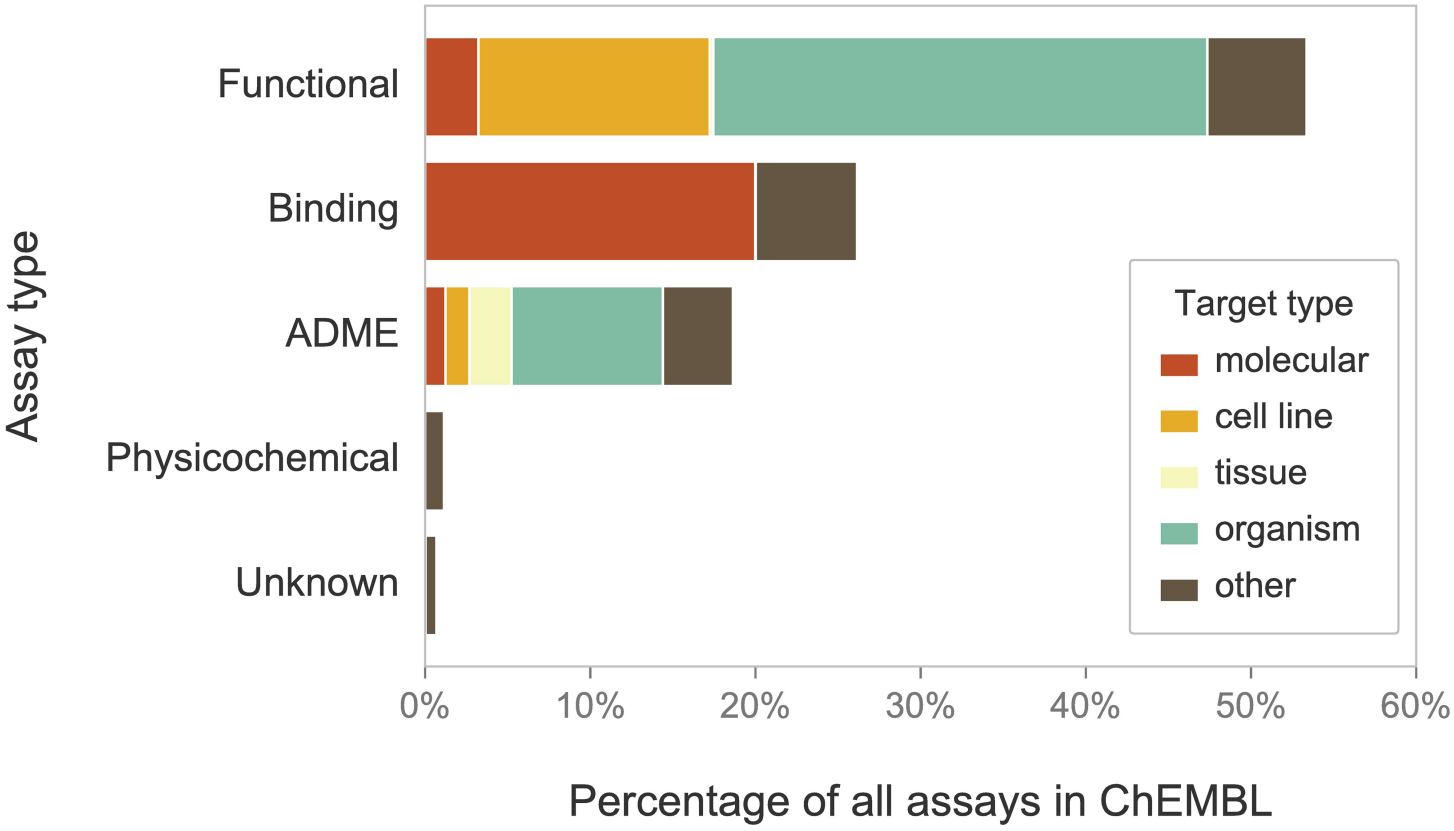
Assay and target type distribution in ChEMBL. Distribution of assay types in ChEMBL (by percentage of all assays in the database) and distribution of the types of associated biological targets. The molecular target category covers multiple ChEMBL target types, including “single protein”, “protein complex”, “protein family”, “nucleic acid”, “macromolecule”, and “protein-protein interaction”.

**Fig 2:**
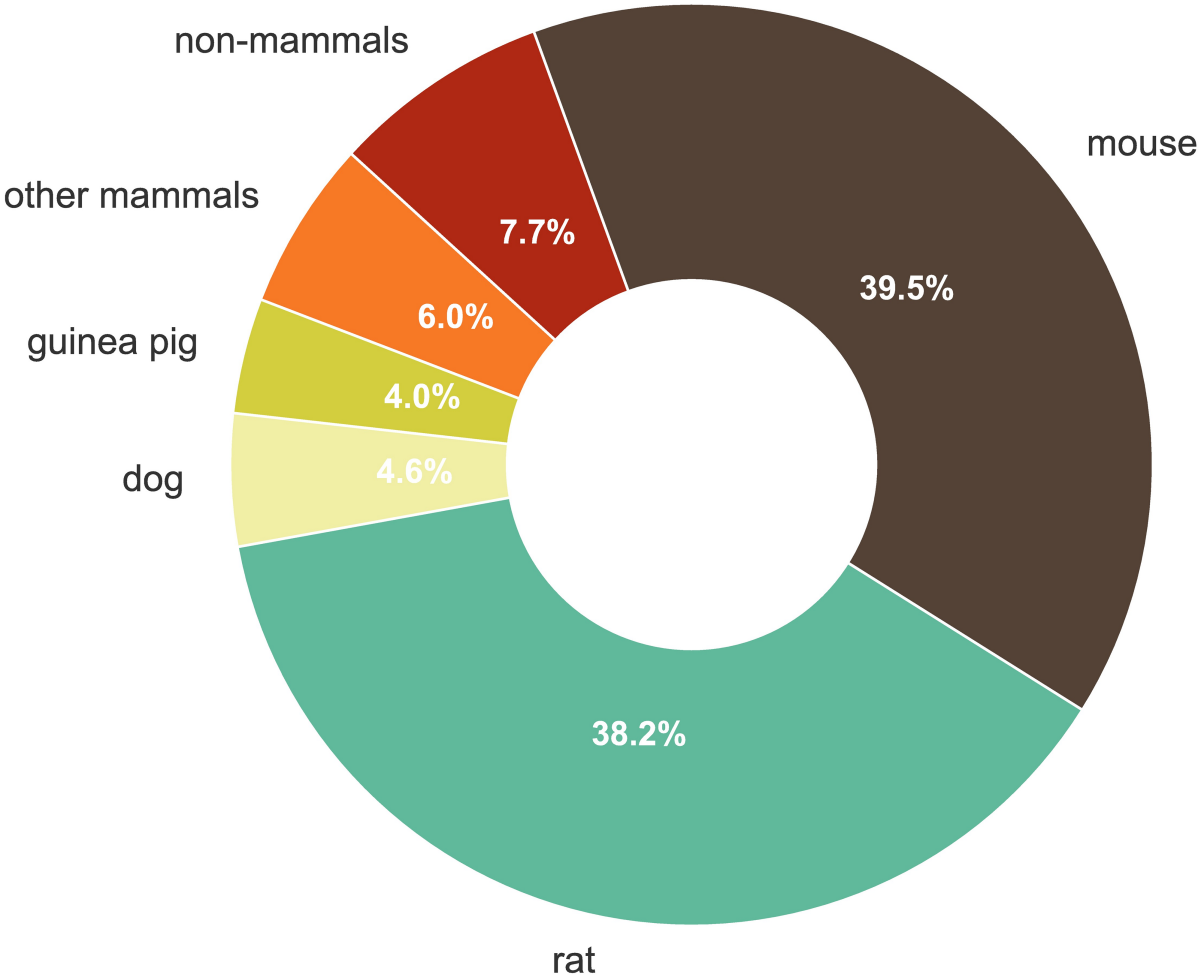
Animals used in *in vivo* efficacy assays. Other mammals include mainly laboratory rodents (*e*.*g*. hamster, gerbil), carnivores (cat), lagomorphs (rabbit), and primates (*e*.*g*. rhesus monkey); the latter were used in 1,157 assays. The main classes of non-mammal animals include arthropods, nematodes, and birds.

ChEMBL is a large open access database covering bioactivity information for about 1.6M compounds tested in 1.2M distinct bioassays. Publications on analysis and use of ChEMBL indicate that most users focus on protein-binding/biochemical data from *in vitro* experiments. However, as shown on Fig 1, more than half of the bioassays in ChEMBL corresponds to “higher-level” functional screening involving cell lines, tissues, and whole organisms. The last category includes laboratory rodents. Rats and mice represent the most commonly studied model organisms in ChEMBL, reflecting their central, historical and current, importance for preclinical drug discovery. Jointly, these two species were used as targets in 100,250 functional assays (77.7% of all animal-based experiments and 84.1% of experiments in mammals); see Fig 2.

**Fig 3:**
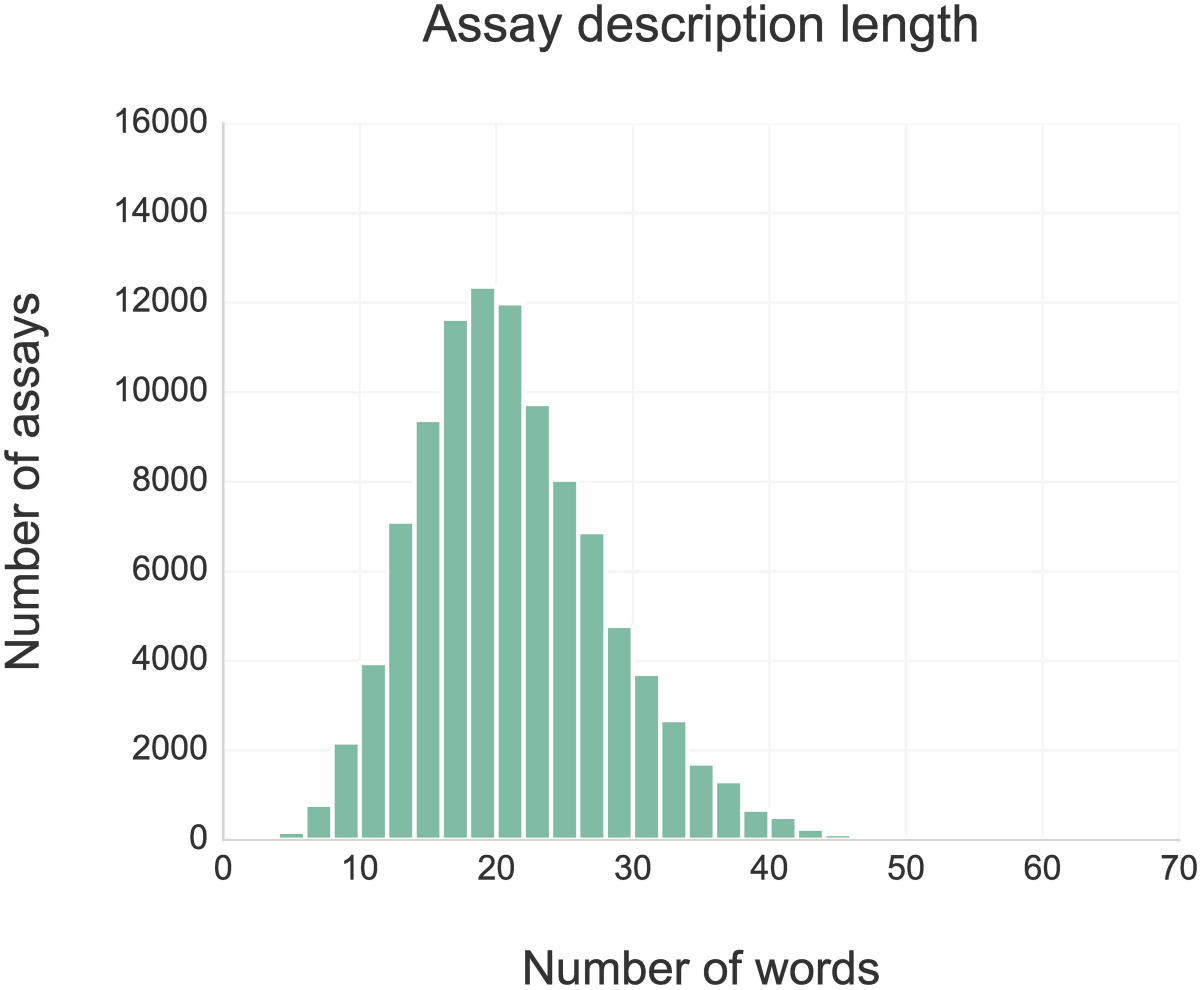
Length of assay descriptions (in words).

**Fig 4:**
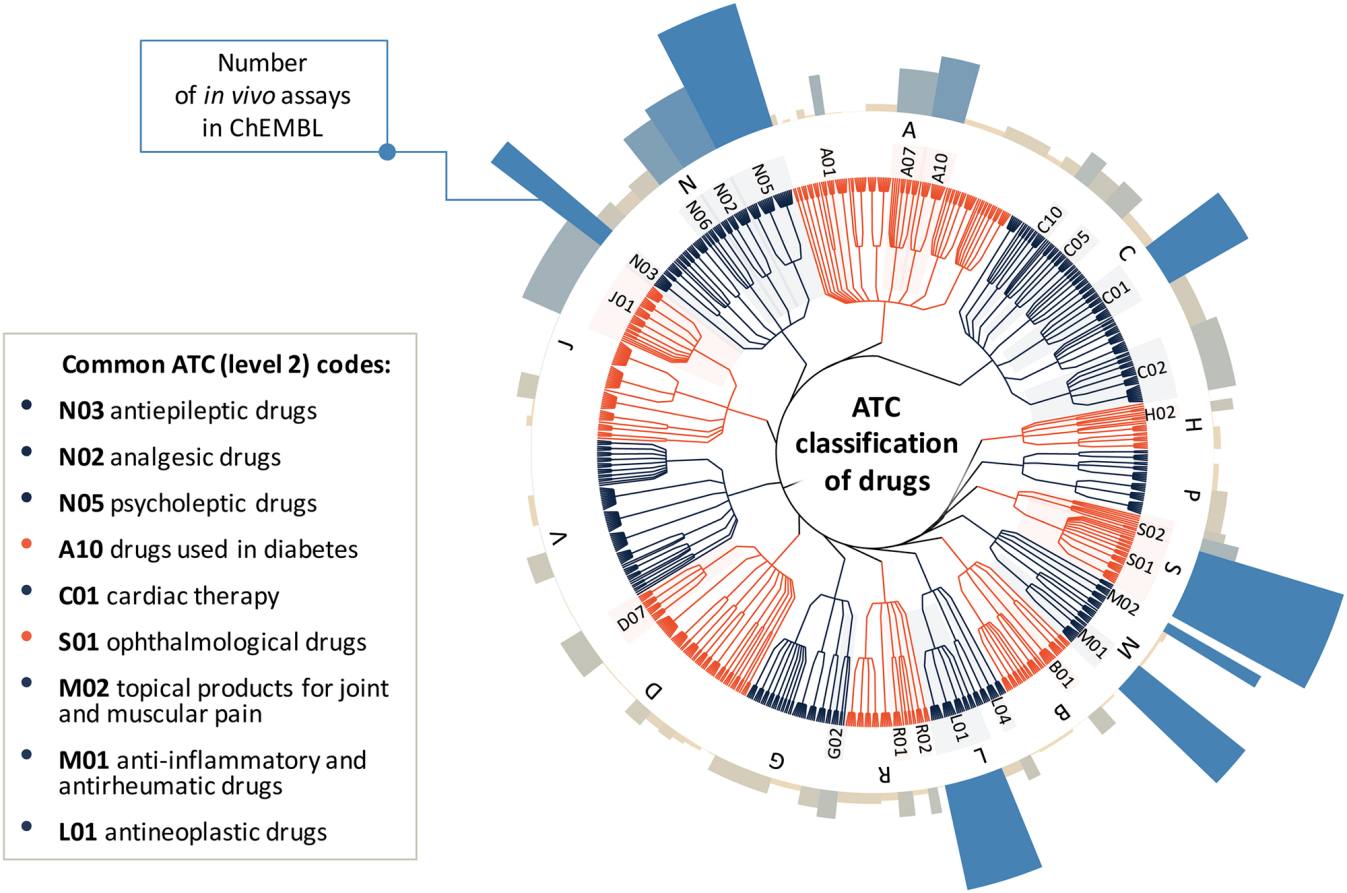
ATC classes of approved drugs tested in *in vivo* efficacy assays. The dendrogram represents the Anatomical Therapeutic Chemical (ATC) drug hierarchy and the coverage of various drug classes in the ChEMBL *in vivo* dataset. The height and color of bars on the circular bar plot (external ring) represent the number of assays involving drugs assigned given ATC code (level 2 of the ATC classification system). Most common ATC level 2 classes corresponding to different therapeutic/pharmacological subgroups are highlighted.

As the input for our analysis, we selected all rat and mouse assay data that were extracted from scientific publications (as opposed to those that came from direct depositions or were loaded from PubChem Bioassay). In summary, the assay data we used come from the full text of 10,851 primary research articles published between 1976 and 2015 in 17 high-impact drug discovery and pharmacology journals. Each assay is summarized by a short description (mean of 20.7, and median of 20 words, see Fig 3) and a set of structured additional annotation fields including species name, molecular structures of compounds tested in the assay, and information about the associated publication including title, year, and journal. Although the total counts of distinct assays performed in rats and mice are similar (49,313 and 50,937 respectively), time frequency analysis shows that mouse is becoming increasingly used in recent years. Altogether, the assays involve 100,432 distinct compounds from all stages of drug discovery, including 1,215 molecules that have at least reached clinical development (based on max phase field in ChEMBL). 19,975 (20% of the total) assays involve approved drugs covering various drug classes and therapeutic areas; see Fig 4 showing the coverage of drug classes defined by Anatomical Therapeutic Chemical (ATC) classification [23].

### Extracting animal model mentions from the descriptions of *in vivo* assays

Following preprocessing of the raw assay descriptions (described in Methods section), the first step in our analysis was to mine the descriptions for phrases representing animal disease models and genetic strains of mice and rats. Currently, there exists no dedicated software for this task although many systems have been developed for the recognition of other biomedical concepts, such as genes, cell lines, and diseases [24–26]. Similarly, there are no labelled datasets that could be used to train supervised machine learning models for animal model identification. Therefore, instead of a supervised approach, we explored dictionary and rule-based methods that make use of structured terminologies, syntactic information, and custom lexical patterns.

To identify genetic strains in text, we built two dictionaries based on specific community nomenclature guidelines [27, 28] and official strain listings maintained by public mouse and rat genome databases where new strains are registered [29, 30]. Each dictionary lists basic strains (1,307 mouse and 648 rat strains), together with the strain type (*e*.*g*. inbred or hybrid), available synonyms, and substrains.

For the task of identification of induced (experimental) disease models, where no such controlled vocabularies exist, we applied a method that identifies relevant expressions using a set of manually defined rules. These extraction patterns combine keyword matching with information about the structure of a sentence to capture animal models, often represented by multi-word phrases such as “maximal electroshock induced” or “high-fat diet-fed”. See Methods section for details on grammatical analysis of assay descriptions and noun phrase extraction.

#### Performance of named entity recognition

With our combined animal model recognition methods, we could identify 1,430 distinct strain, transgenic and induced animal model names in 57,538 assay descriptions. We evaluated the performance of our approach against a dataset of 500 randomly selected assay descriptions manually annotated by two curators. The strict/relaxed interannotator agreement was 89.8%/93.6% for animal models and 77.3%/85.4% for phenotypes. Table 3.4 in S1 Text reports the performance measures for four tasks: detection of genetic strains, experimental models, transgenic animals, and phenotypes. The F-score, calculated based on exact/partial matches, reached 0.95/0.96 for genetic strains (based on 183 occurrences in the benchmark set), 0.83/0.88 for experimental models (316 occurrences), 1.0/1.0 for transgenic models (13 occurrences), and 0.61/0.76 for phenotypes (273 occurrences) respectively; see S1 Text for details, comparisons, and misannotation examples. Typically, the precision was higher than recall—particularly in the case of genetic strain detection, where a dictionary-based method was used. The low occurrence of transgenic model mentions in the annotated dataset reflects the relative scarcity of this class of animal model bioassays in ChEMBL and, hence, across the broader drug discovery literature.

#### Frequency analysis of extracted terms

**Fig 5:**
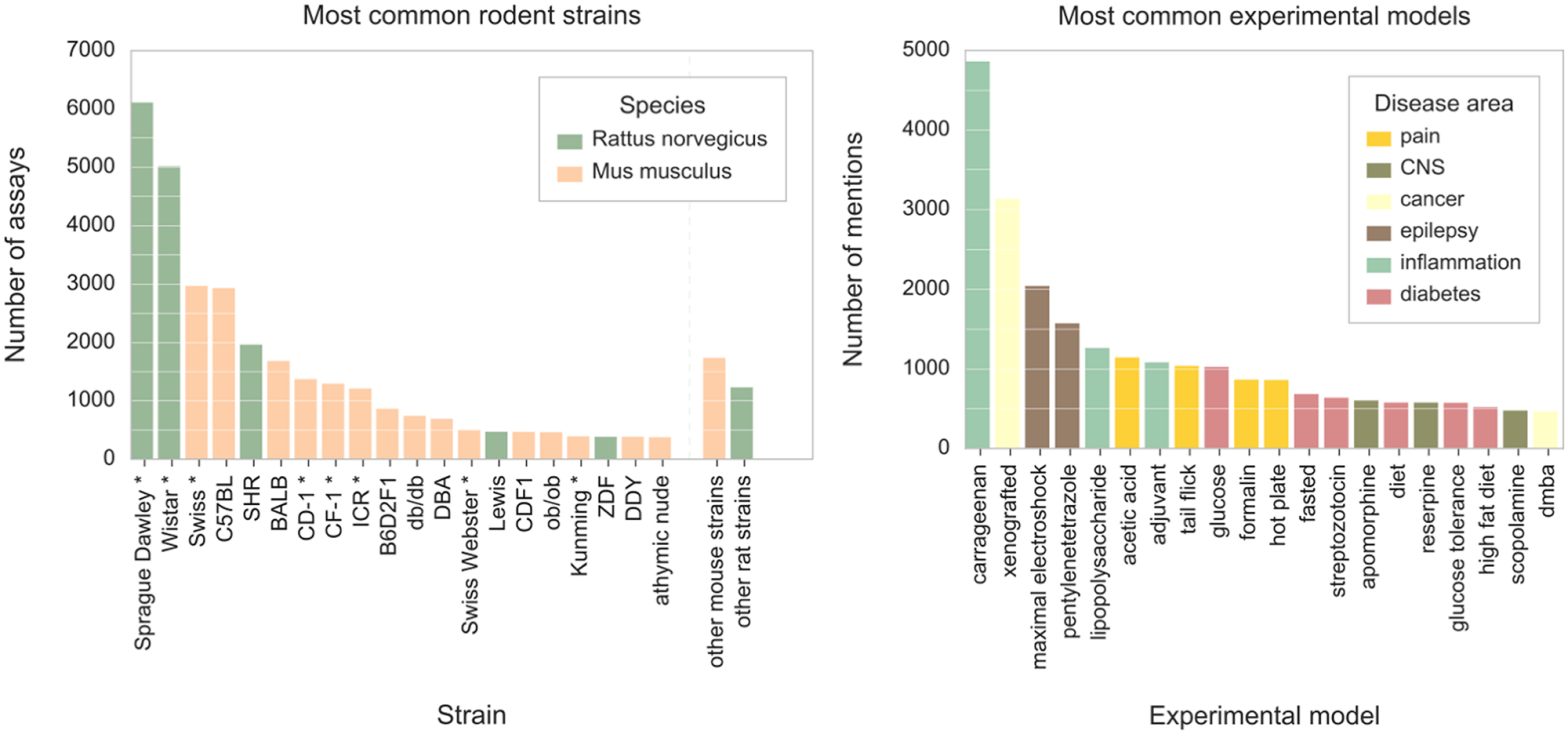
Most common rodent strains and experimental disease models mentioned in the descriptions of *in vivo* efficacy assays in ChEMBL. **(A)** Twenty strains that are most frequently mentioned in assay descriptions; outbred strains are marked with an asterisk (*). Upon identification in the text of assay descriptions, the strain names were normalized using strain synonym listings maintained by rodent genome databases. For instance, C57BL mouse was described in various descriptions with more than 30 different terms including names that do not follow official nomenclature guidelines: “BL6”, “Black6”, or “C57/Black”. **(B)** Bar plot showing twenty experimental models that are most frequently mentioned in assay descriptions. The models were manually annotated with disease area.

Many of the strains that are frequently mentioned in the assay descriptions (Fig 5A) involve spontaneous (genetic) models used to study specific diseases, particularly hypertension, diabetes, and cancer. For instance, spontaneously hypertensive rat (SHR) was used in more than 1,960 assays whilst three most common spontaneous models of diabetes (ob/ob mouse, db/db mouse, and ZDF rat) were mentioned 1,575 times. However, as further discussed in the next section, the most commonly used strains are long-established general-purpose models including many outbred strains such as Sprague Dawley rats and Swiss mice whose genetic background is diverse and non-uniform.

Amongst the experimental animal models used in ChEMBL assays (Fig 5B), the most common are models of inflammation, epilepsy, pain, and cancer, whose conditions were induced using a variety of experimental stimuli including chemicals (*e*.*g*. in carrageenan model), tumor transplants (xenograft models), electrical stimuli (maximum electroshock seizure (MES) model), and thermal stimuli (hot-plate test).

In addition to animal models, we identified the phenotypic and behavioral terms in the descriptions using a dictionary-based method leveraging existing ontologies. Here, the most common detected terms correlate with the therapeutic use (indications) of the disease models: edema, seizures, life span, leukemia, arthritis, *etc*. Finally, the most frequent behavioral terms were “nociceptive behavior”, “writhing”, and “licking”–behaviors observed and measured in animal models of pain.

### A semantic space of animal models and phenotypes

Our next goal was to automatically organize and cluster concepts extracted in the previous step. This involved finding related entities—such as animal models of the same disease—identified based on linguistic patterns and contexts in assay descriptions. The underlying assumption is that two terms are likely to be related if they often occur in similar contexts, *i*.*e*. surrounded by the same or analogous phrases [31].

To find semantic similarities between words/phrases, we used the assay descriptions to train Word2Vec [32]–an unsupervised neural network (NN) model that converts text into a set of numerical vectors. These word vectors, also called word embeddings, correspond to points in a high-dimensional semantic space where distance correlates with differences in meaning. In other words, vectors representing semantically related words lie close to each other in the constructed semantic space, while unrelated words are far apart [33].

#### Semantic similarities between concepts

We trained a Word2Vec model with the corpus of preprocessed *in vivo* assay descriptions—following shallow parsing and noun phrase extraction workflow summarized in Methods section. As output, we obtained a set of 250-dimensional numerical vectors (word embeddings), each corresponding to a single word (*e*.*g*. “analgesia”) or multiword phrase (*e*.*g*. “arterial pressure”).

Simple mathematical measures, such as a Cosine vector similarity, can be used to quantify distances between the word embeddings [34]. Among other tasks, these can be used to exploit the semantic neighborhoods of interesting concepts to find related words and phrases. Table 1 shows examples of such semantic similarity calculations for several query terms. As shown in the table, Word2Vec places synonyms close to each other: “antihyperglycemic activity” and “hypoglycemic activity” have a very high cosine similarity just as “pentylenetetrazole induced” and “ptz induced” (where PTZ is a commonly used acronym for pentylenetetrazole). This is also true for non-synonymous words used in the same context. For instance, the most similar terms to “heart rate” include “arterial pressure” and “systolic blood pressure”–other common biomarkers used in assays testing drugs for cardiovascular indications, whilst terms like “L1210 leukemia” correspond to names of different cancer cell lines used in mouse xenograft studies.

**Table 1:** Example Word2Vec queries. For each example query, the table shows four most similar words/phrases as measured by Cosine similarity of associated vector embeddings (shown for each result). The embeddings were learned by Word2Vec model trained with preprocessed *in vivo* assay descriptions (following shallow parsing and noun phrase extraction workflow summarized in the Methods section).

**ZDF rat**	**Pentylenetetrazole induced**	**Reserpine induced**
db/db mouse (0.914) zucker fatty rat (0.898) ob/ob mouse (0.894) KKaY mouse (0.888)	ptz induced (0.938) maximum electric shock induced (0.886) subcutaneous pentylenetetrazole induced (0.862) picrotoxin induced (0.862)	tetrabenazine induced (0.839) WIN 55,212–2 induced (0.838) 8-OH-DPAT induced (0.838) haloperidol induced (0.828)
**brain**	**antihyperglycemic activity**	**analgesic activity**
striatum (0.844) frontal cortex (0.826) cerebellum (0.802) hippocampus (0.789)	antidiabetic activity (0.947) diabetic assay (0.931) antihyperlipidemic activity (0.913) hypoglycemic activity (0.908)	antinociceptive activity (0.952) analgesia (0.781) analgetic activity (0.759) nociception (0.741)
**convulsion**	**L1210 leukemia**	**heart rate**
convulsions (0.952) seizure (0.940) seizure assay (0.933) clonic seizures (0.905)	B16 melanoma (0.842) L1210/ARA-C leukemia (0.838) P388 leukemia (0.827) M5076 reticulum cell sarcoma (0.820)	arterial pressure (0.923) systolic blood pressure (0.897) diastolic blood pressure (0.865) blood pressure (0.855)

Importantly, many of these terms are present in ontologies and term associations established by Word2Vec often reflect the ontological relationships. However, this is not always the case. For instance, two terms representing common behavioral endpoints measured in pain assays: “writhing” and “abdominal constriction” are predicted to be very similar in meaning by the Word2Vec model. Whilst the first one is present in the Neuro Behavior Ontology, the latter is not; in fact it is not present in any of the 525 biomedical ontologies covered by National Center for Biomedical Ontologies, NCBO [35]. Such results suggest that a set of vectors learnt by a well-trained Word2Vec model could support the task of ontology expansion [36].

#### Analogy queries

In addition to similarity queries based on simple distances in high-dimensional semantic space, more complex geometry-inspired relationships between words can be calculated to uncover analogies in the data. Mathematically simple calculations—sums and subtractions performed on word vectors—can sometimes uncover surprising semantic regularities in the documents [34]. A classic example of such semantic analogy is the following relationship between four words: king—man + woman = queen [34]. In this study, we observed many examples of such complex relationships. For example, the model predicts that “insulin level” is to “Zucker rat” (a spontaneous model of diabetes) what “heart rate” is to “SHR rat” (a model of hypertension). Although meaningful semantic analogies were limited to the well-represented words in the assay descriptions, it is nevertheless encouraging that the model can uncover such complex word relationships based on mere contextual information and without any human supervision or bias.

#### Clustering animal models and phenotypes

In the next step, rather than searching the entire semantic space for similar terms, we selected a set of interesting concepts and used the model to find the semantic relationships between them. Specifically, we calculated pairwise similarities between word embeddings of frequently mentioned animal models and phenotypes, and used these similarities as input for hierarchical clustering.

**Fig 6:**
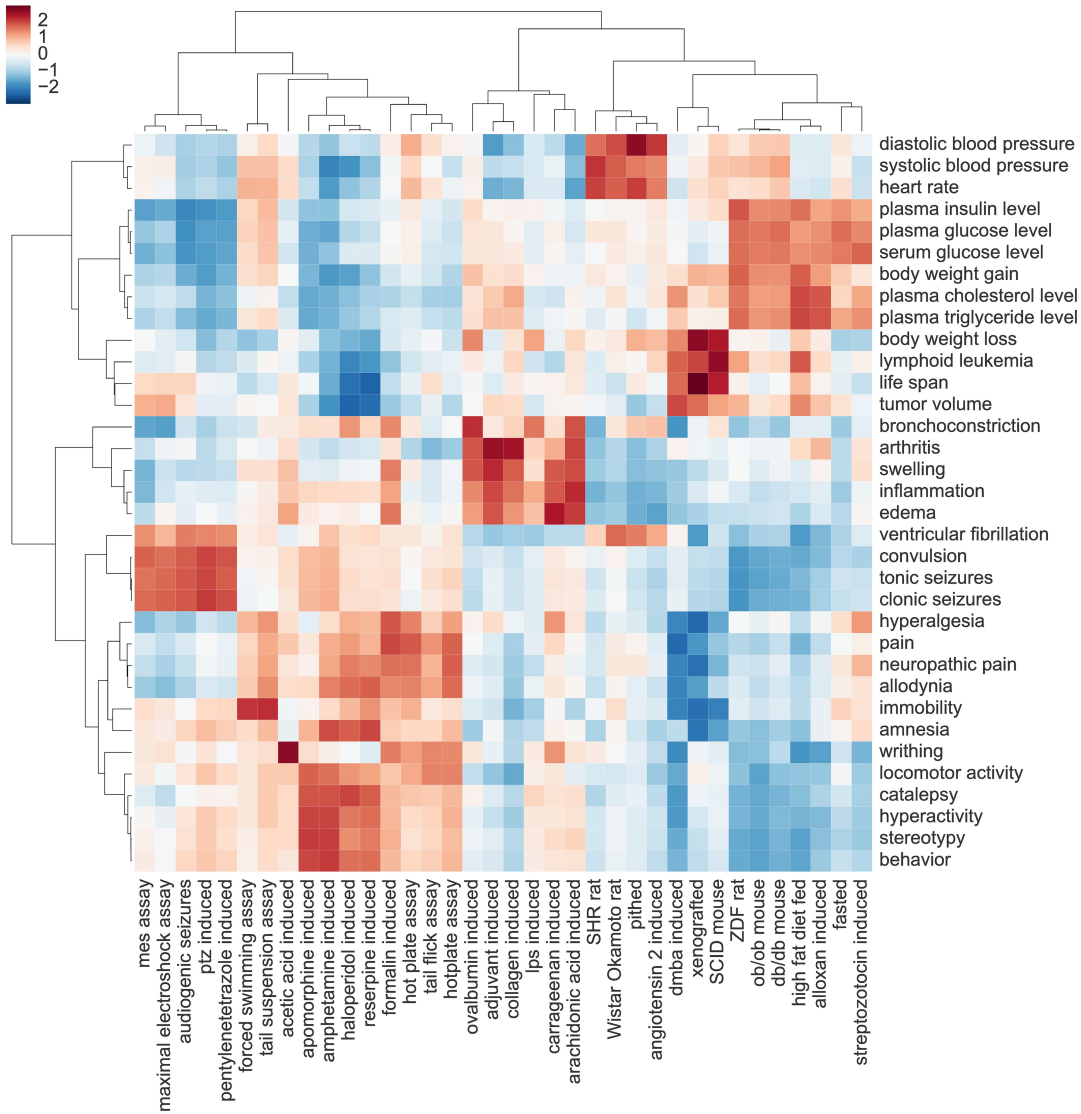
Semantic similarities between animal models and phenotypes. A hierarchically clustered heatmap showing pairwise semantic similarities between 35 animal models and 35 phenotypes frequently mentioned in the assay descriptions. Red color corresponds to higher, blue—to lower semantic similarity; values in each row are Z-score normalized. Both rows and columns are hierarchically clustered (using average linkage and Euclidean distance) and the results are represented as dendrograms. Semantic clusters, shown as red regions on the heatmap, correspond to distinct disease areas including epilepsy, pain, inflammation, hypertension, diabesity, and cancer. The figure provides an automatically-generated summary of the use of common animal models to study the effect of drugs on different types of disease-related phenotypes.

The resulting heatmap is shown on Fig 6. Of note, related animal models and phenotypic terms tend to group together around distinct disease areas; this is shown by the side dendrograms and red regions of the heatmap. For instance, biomarkers of hypertensions, such as heart rate or diastolic blood pressure, cluster with animal models of hypertension. The latter include both spontaneous and induced animal models: spontaneously hypertensive rat (SHR)–a genetic model of hypertension, lies very close to an experimental model of the same disease in which hypertension is induced by infusion of angiotensin. Other disease areas represented by the clusters on the heatmap are epilepsy, hypertension, cancer, pain, inflammation, and diabesity (obesity and diabetes combined). Meaningful clustering of animal models and phenotypes shows that Word2Vec can be used to effectively organize and summarize entities extracted from text.

### Classification of *in vivo* assays

Next, we considered the problem of assay classification. Specifically, we tested whether features learnt by Word2Vec could be used to group together related *in vivo* assays and to predict the type of an assay based on its textual description. To train supervised classification models, we used the Word2Vec embeddings labeled with the curated information associated with compounds tested in individual assays. Specifically, many assays in ChEMBL involve well-known reference molecules (pharmacological standards/positive controls) used to calibrate and validate the resulting measurements on novel molecules. Commonly, such molecules are comparator approved drugs, whose biological activities and therapeutic effects are already well studied. These characteristics are summarized by Anatomical Therapeutic Chemical (ATC) classification codes—the most widely recognized drug classification system administered by the World Health Organization [23]. Here, we used ATC codes of involved drugs to link assays to various disease/therapeutic area indications.

#### Construction and visualization of a semantic space of assay descriptions

We selected a subset of 19,975 (approx. 20%) of animal-based assays that involve approved drugs, and used the level 2 ATC codes assigned to those drugs to label assays and divide them into classes (see below). We then converted assay descriptions into computable representations—numerical vectors that would serve as input for the classification models. We calculated those by averaging the individual word embeddings generated for each assay description by the Word2Vec model. In this way, we constructed a new semantic space, where each point corresponds to an entire assay description rather than to an individual term.

**Fig 7:**
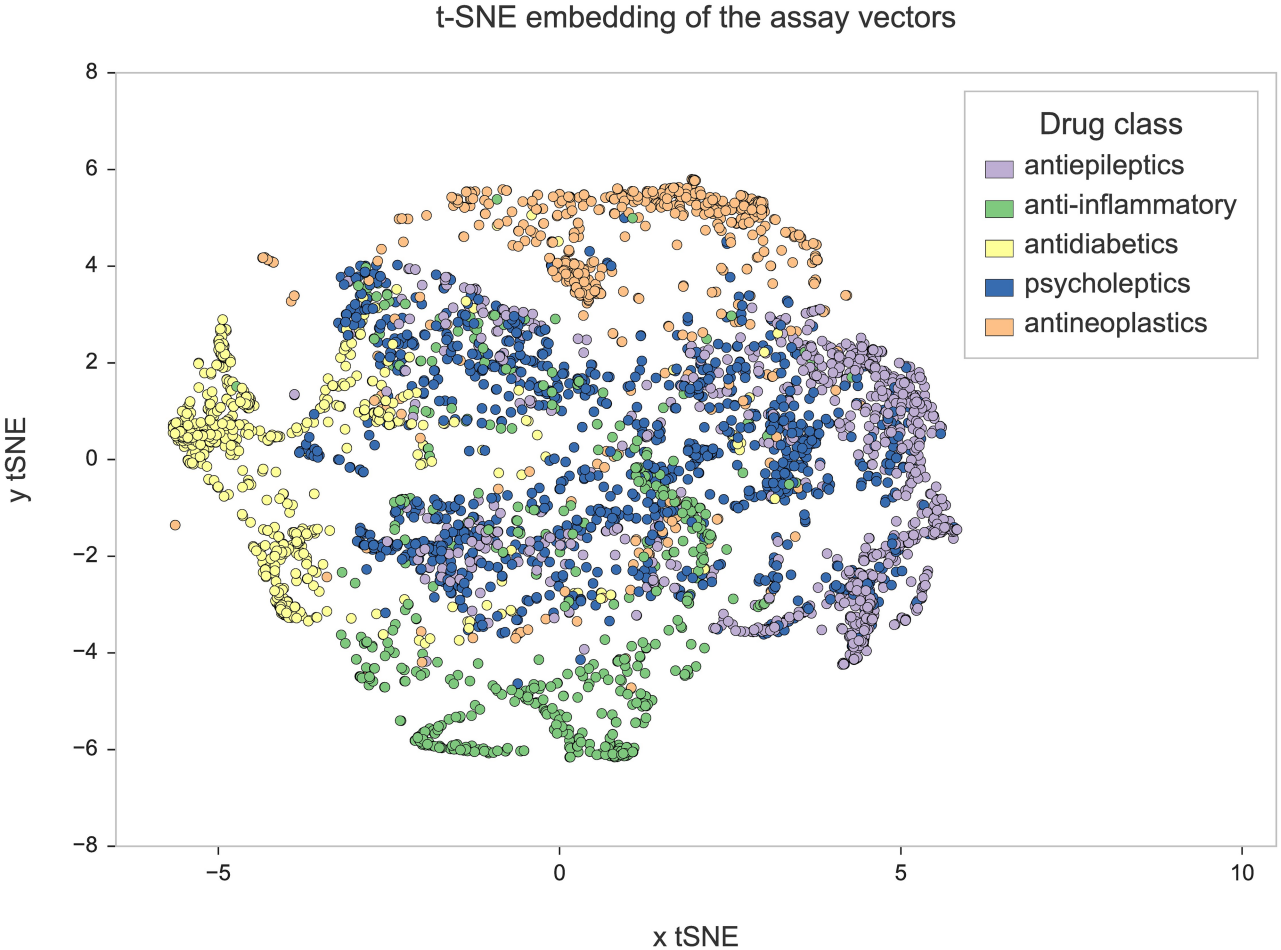
Visualization of a semantic space of assay descriptions. Vector representations calculated for individual assay descriptions were projected into two-dimensional space and visualized as points on a scatterplot. The colors correspond to ATC codes of approved drugs tested in the assays: antiepileptics, N03; anti-inflammatory, M01, M02, C01, S01; antidiabetics, A10; psycholeptics, N05; antineoplastics, L01.

To qualitatively analyze the distribution of assays in this semantic space, we projected the vectors into 2D using t-SNE—a dimensionality reduction technique based on Stochastic Neighbor Embedding [37]. We then visualized the subset of assays which involved drugs from 5 most common classes (five top ATC level 2 codes): antiepileptic, psycholeptic, antineoplastic, antidiabetic, and anti-inflammatory drugs (see Methods section for details). As shown in Fig 7, the assays involving drugs with the same ATC codes are clustered in distinct regions of the plot. Since the vectors used in visualization are based purely on semantic features this result shows that the descriptions of assays testing the same drug class use similar vocabularies and that Word2Vec can preserve this similarity when converting text into numerical vectors. Indeed, whilst descriptions of assays testing anti-inflammatory drugs are likely to mention “edema”, “swelling”, “acute pain”, “carrageenan”, or “paw volume”, assays testing antidiabetic drugs are more likely to include such phrases as: “antidiabetic activity”, “blood glucose level”, and “body weight”. For more examples, see Table 2, which reports top five enriched words (ranked by a simple Fisher test p-value) as well as most frequent drugs, animal models, and phenotypes for each of the five categories.

**Table 2:** Most common drugs, phenotypes, experimental animal models, and top 5 enriched phrases (ranked by a simple Fisher test p-value) for the five most common ATC combinations. Phenotypes, animal models, and noun phrases were text-mined from the text of assay descriptions; drug names were extracted from structured data fields in ChEMBL.

Drug class	Drugs	Phenotypes	Experimental models	Phrases
**antiepileptics**	phenytoin carbamazepine phenobarbital ethosuximide valproic acid	seizures shock nociceptive behavior pain clonic seizures	maximal electroshock pentylenetetrazole rotarod test bicuculline CCI	protection anticonvulsant activity intraperitoneally TD50 protective
**psycholeptics**	diazepam haloperidol clozapine chlorpromazine risperidone	behavior process seizure catalepsy stereotypic behavior locomotor activity	apomorphine pentylenetetrazole amphetamine rotarod test pentobarbital	climbing antipsychotic activity anxiolytic activity neuroleptic activity stereotypy
**antineoplastics**	cytarabine doxorubicin mitomycin fluorouracil tiazofurin	leukemia neoplasm life span trait mortality/aging body weight	P388 leukemia L1210 leukemia Lewis lung carcinoma Ehrlich’s ascites B16 melanoma	antitumor activity survivors day inoculated implanted
**antidiabetics**	rosiglitazone glyburide metformin tolrestat plioglitazone	blood glucose level plasma glucose level cataract body weight triglyceride amount	glucose tolerance test streptozotocin glucose challenge fasted alloxan	antidiabetic activity antihyperglycemic activity diabetic reduction RvB
**anti-inflammatory**	indomethacin phenylbutazone rofecoxib zomepirac ketoprofen	edema arthritis swelling hyperalgesia inflammation	carrageenan adjuvant acetic acid TPA yeast	anti-inflammatory activity paw edema inhibition paw oedema acute anti-inflammatory activity

#### Assay classification models

In addition to simple visualizations, we also used the assay vectors to build and evaluate several random forest classifiers. We considered four different classification problems, each based on ATC codes of approved drugs tested in the assay (see: Methods section). For instance, one classifier was trained to predict whether an assay involved any drugs acting on nervous system whilst another model classified assays based on a specific subclass of such drugs: “psycholeptics”, “antiepileptics”, “antiparkinsonians”, *etc*. In each case, we applied two different methods for dividing the data into train and test sets (assay and document-based split) to avoid overestimating the prediction accuracy due to the similarity of assay descriptions curated from the same scientific article [38] (see Methods section for details).

**Fig 8:**
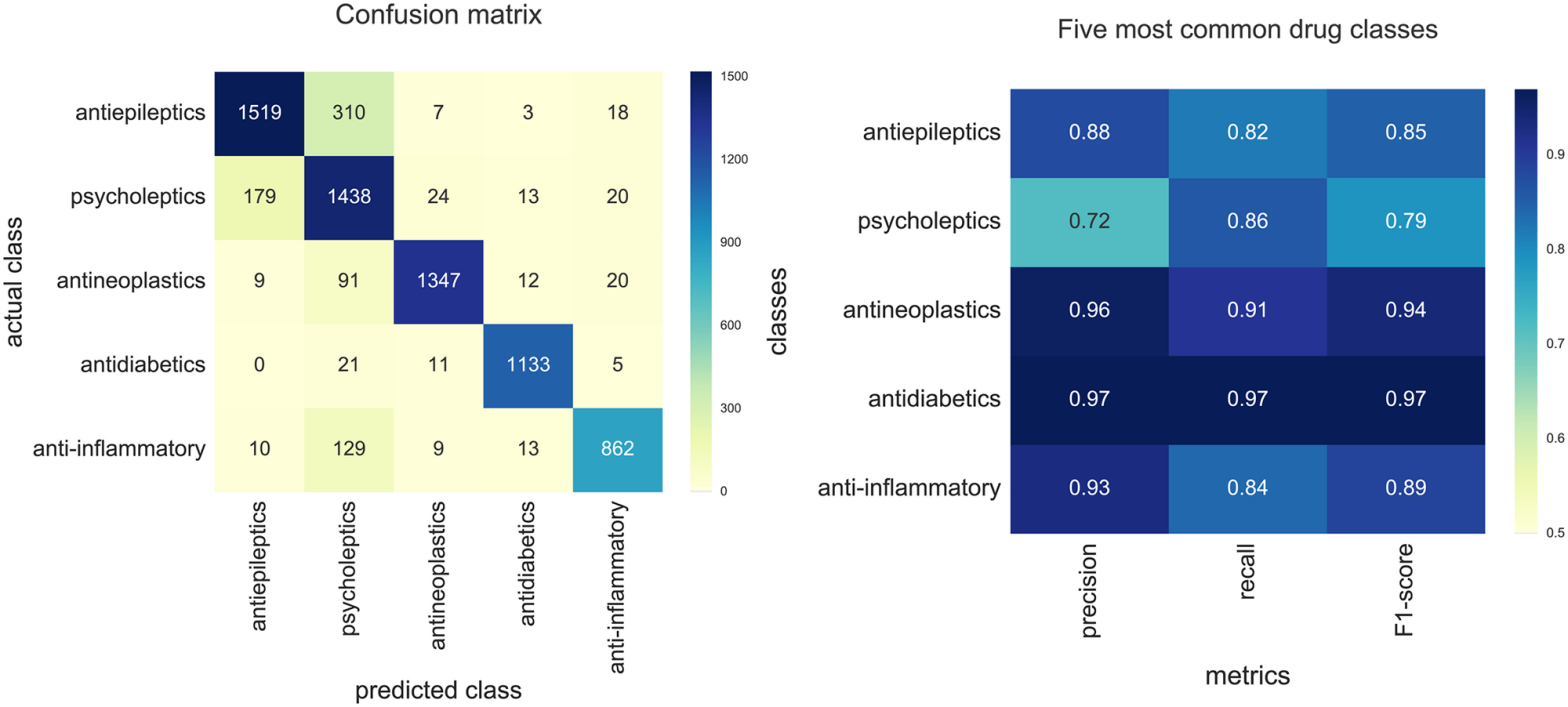
Confusion matrix and per-class performance measures calculated for one of the random forest classifiers. The figure shows performance measures calculated for a multiclass random forest classifier that assigns each assay with one of the five most common ATC code combinations—a proxy for the most common disease areas in ChEMBL. The model was built with data visualized on Fig 7; strict partitioning method based on random document split was used to partition the dataset into cross-validation subsets. The model achieved overall prediction accuracy of 0.87. (A) Per-class confusion matrix. (B) Per-class classification report.

Following 10-fold cross-validation procedure, we calculated the overall prediction accuracy and per-class performance measures for each classification problem. Fig 8 shows classification report and confusion matrix for an example classifier while all the results are detail in S1 Text. We found that all classifiers achieved high overall performance in predicting ATC classes even though no chemical structure information was used to train them and assay descriptions do not normally contain any drug information. As further reported in S1 Text, the classifiers based on assay vectors outperformed two other text-based approaches: paragraph2vec and bag-of-words with TF-IDF weighting. One of the assay vector-based classifiers could distinguish between assays involving cidal/cytotoxic drugs (such as anticancer and antimicrobial drugs) and non-cytotoxic drugs with high overall accuracy of 0.97 (or 0.92 for document-based split). Another classifier predicting whether an assay involved any drugs acting on nervous system achieved overall accuracy of 0.93 (or 0.86 for document-based split); the classifier predicting the specific subclasses of such drugs had the lowest, but still potentially useful accuracy of 0.86 (decreasing to 0.67 when the document-based test-training partitioning method was applied).

#### Analysis of misclassified assays

The prediction performance varies considerably between different models as well as between specific assay classes considered within a single model. These differences might be explained by the complexity of the model (*e*.*g*. multiclass vs binary classifiers), size of the dataset used in training, and dataset imbalance; see S1 Text. For instance, there are ten times more assays involving antiepileptic drugs than there are assays involving anesthetics, reflecting the relative historical efforts in the development of drugs for particular therapeutic applications. This leads to an unbalanced training set, and consequently, the recall value calculated for the latter class was much lower. The analysis of specific misclassified assays showed that some errors were due to very short, non-informative assay descriptions (“Activity assessed as weight loss”) or due to the variation in the ATC codes assigned to a single drug.

In addition, some misclassified assays (according to our label assignment detailed above) involved drugs tested for therapeutic activity beyond their approved indication. This category included several interesting examples. For instance, one misclassified ChEMBL assay (CHEMBL994451) involved Gemfibrozil—a lipid-modifying medicine approved for the treatment of hyperlipidemia. The assay was erroneously assigned by the model to the “cidal” class grouping anticancer and antimicrobial drugs. However, manual inspection revealed that the data have been extracted from a drug repurposing study in which the authors reported increased survival of influenza-infected mice treated with Gemfibrozil and proposed that the drug could be adapted for the use against the viral infection [39].

In another case, several assays involving an anti-HIV medication, Zidovudine were assigned to the “non-cidal” class by our model and, hence, were detected as misclassified. These assays, however, did not evaluate the well-known antiviral properties of the drug, but its impact on cholesterol and insulin levels related to the lipodystrophy syndrome side effect observed in HIV-infected patients receiving the treatment [40]. Therefore, the property tested in the “misclassified” assays was not the therapeutic effect of the drug, but, in fact, its potential side effects.

### Linking animal models to other biological entities

To better understand how animal models were used in drug discovery, we have combined the results of our text mining analysis with the manually abstracted content of ChEMBL. Specifically, we used the curated information about compounds evaluated in each *in vivo* assay to link animal disease models with approved drugs that had been tested in them. Based on the relationships generated this way, we built and visualized a network representation of the ChEMBL *in vivo* data.

**Fig 9:**
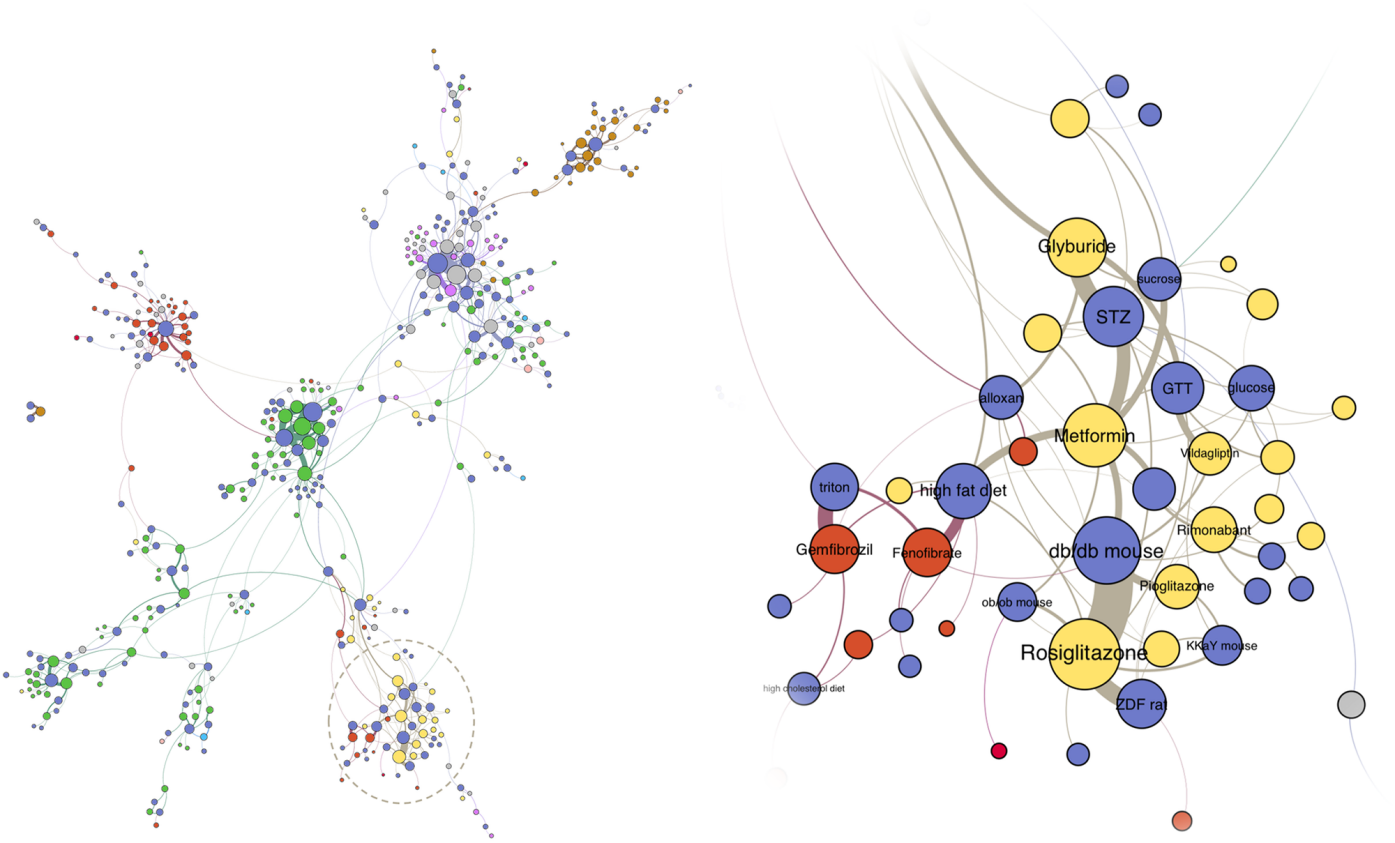
Major component of the animal model—Drug network with detailed “diabesity” cluster. The nodes in the graph correspond to approved drugs and animal models of disease, including induced, spontaneous, and transgenic disease models text-mined from assay descriptions. A drug is linked to an animal model if it was tested in at least five assays involving this model. Drug nodes are colored according to the assigned ATC (level 2) codes, while animal model nodes are blue; node size is proportional to the number of assays involving a given drug or model. Animal model-drug relationships visualized in the graph are listed in the S5 Dataset. STZ, streptozotocin-induced model; GTT, glucose tolerance test; ZDF, Zucker Diabetic Fatty rat; glucose, glucose-loaded model.

As shown on Fig 9, the drug-model network shows strong local clustering with several densely-connected modules that correspond to distinct disease classes including inflammation, cancer, epilepsy, and hypertension. The clusters bring together related drugs and animal models of related diseases. For instance, in the highlighted cluster, antidiabetic drugs (such as metformin or rosiglitazone) and lipid-modifying agents (*e*.*g*. gemfibrozil, fenofibrate) are connected through various animal models used in diabetes and obesity research. The disease models grouped in the cluster belong to different classes. Some of the models include diabetic animals whose condition was artificially induced in the laboratory: either through adjusted diet (e.g. high-fat diet and glucose load models) or through administration of toxic compounds such as streptozotocin (STZ) or alloxan—chemicals that destroy pancreatic cells thus dramatically reducing insulin production [7]. In addition to the experimental models, several spontaneous (genetic) models can be found in the cluster. For instance, *db/db* mice and Zucker Diabetic Fatty (ZDF) rats develop symptoms similar to human diabetes due to a mutation in the *Lepr* gene, which encodes the receptor for a “satiety hormone”, leptin [7]. Finally, one of the smaller nodes in the network corresponds to a transgenic model: a genetically engineered mouse model expressing high levels of human apolipoprotein A-I (*APOA1*).

**Fig 10:**
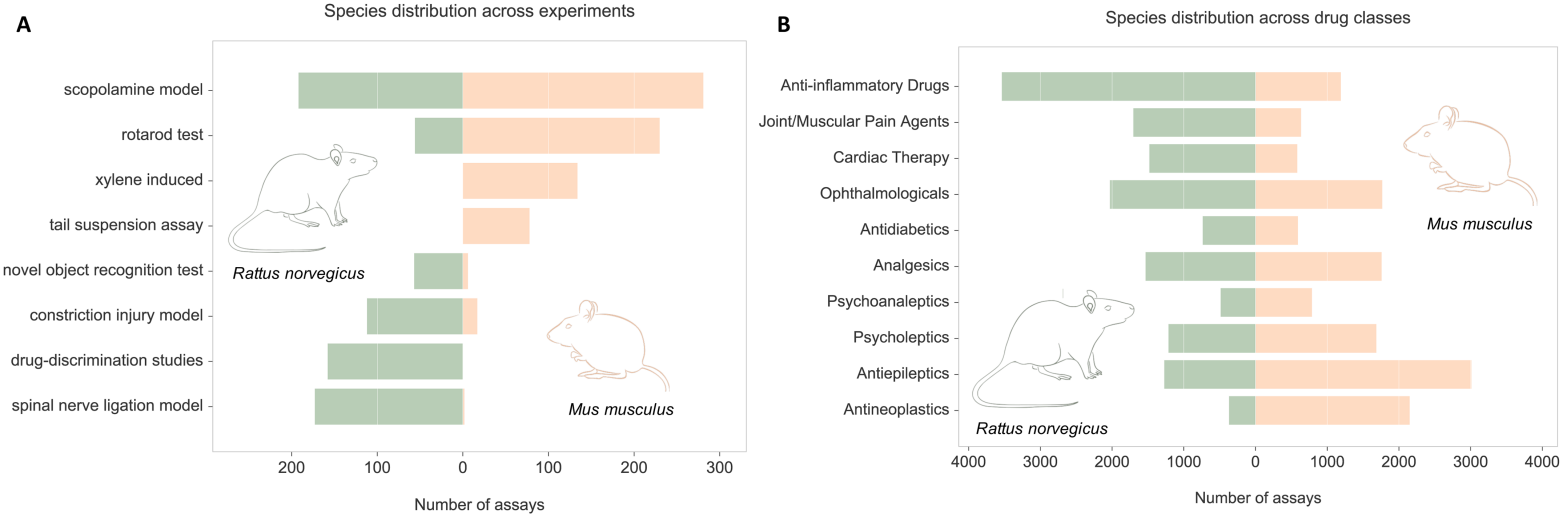
Differential use of rats and mice across *in vivo* assays. **(A)** Number of assays involving rats and mice for eight example experimental systems. **(B)** Differential use of the two rodent species in assays testing drugs from the 10 most common drug classes (based on the second level of the ATC classification). Classes are ordered by the difference in the number of assays involving rats and mice. The images of the animals used in the figure were obtained under the open license from Gene Expression Atlas https://www.ebi.ac.uk/gxa.

In addition, we combined the extracted animal model information with curated species annotations from ChEMBL to investigate differential usage of mouse and rats in drug discovery. First, we compared organism annotation across all assays involving different experimental models; representative results are shown on Fig 10A. Next, we divided assays into classes corresponding to different disease areas (based on the ATC codes of involved approved drugs) and found species distribution for the most frequent indications (Fig 10B). The figures show that whilst some screening experiments are routinely performed on animals of different species, in some cases the rat might be preferred to mouse, or vice versa. As further discussed in the next section, the variation may be attributed to various factors such as anatomical and behavioral differences between the two rodents.

## Discussion

The ChEMBL *in vivo* data, which capture in an unbiased way reported bioactivities from animal-based drug screening experiments, are arguably the database’s least appreciated resource. However, there are several reasons why the dataset could be valuable for data-driven translational drug discovery research. Firstly, the ChEMBL *in vivo* data are unique: there are almost no other large publicly available resources for efficacy screening in animal models. Secondly, they have been derived from scientific articles published over the last forty years and, hence, they reflect long-term, community-wide trends in preclinical drug discovery. Finally, the information from these publications has already been extracted, filtered and condensed by specialists and trained curators. In some sense, it is more efficient to analyze the concise curated summaries resulting from these efforts than to attempt to mine the original publications directly.

To our knowledge, this is the first systematic analysis of the *in vivo* assay description data in ChEMBL and the first attempt to index and identify spontaneous and induced animal models from such a resource.

### Mining and classifying *in vivo* assay descriptions

Although most information on the *in vivo* assays in ChEMBL is in the form of unstructured assay descriptions, we show that it can be efficiently extracted and systematized using modern text mining techniques. In this work, we first identify mentions of animal models and phenotypes, and automatically organize them using a neural network language model. Then, we use the learned neural embeddings (word vectors) to train a random forest classifier predicting ATC codes of drugs tested in different assays. In the following, we further discuss our approach as well as its potential applications and limitations. In addition, we examine how the information extracted from assay descriptions reflects trends and practices in preclinical drug discovery.

#### The semi-standardized format of the assay descriptions facilitates the construction of a meaningful semantic space

In the first experiment, we used Word2Vec model to construct a multidimensional semantic space of words and phrases used in the assay descriptions. We then analyzed the structure of this space with a focus on semantic similarities between extracted biological concepts. The results show that animal models and phenotypes related to the same disease tend to cluster together, suggesting that the model can capture real functional similarities, and innate relationships within the domain.

Since the algorithm depends on a variety of contexts and subtle variations in vocabulary, the results indicate that there is enough information encoded in the assay descriptions to enable a meaningful analysis—particularly in the case of well-represented concepts and the more frequently studied therapeutic areas. Although unsupervised language models like Word2Vec normally require large training sets for high quality predictions [33], the model presented here can automatically identify related biological concepts, despite the comparatively limited size of the ChEMBL *in vivo* assay description dataset.

We attribute this performance to the consistent domain and format of the sentences that the model was trained on. Although intended for humans, not algorithms, many assay descriptions might be considered semi-standardized, or “canonical”, since they have been written by a relatively small and discrete set of humans following certain curation and operational guidelines. We deduce that the experienced curators tend to use standard terminology and similar grammatical patterns when manually extracting data for ChEMBL. This leads to more linguistic regularities that computational models depend on [41]. Moreover, the concise format of assay descriptions leads to high information density and little irrelevant content that could otherwise obscure the signals detected by the algorithms.

In addition, we observed that the performance was improved by our multistep text processing workflow. While most standard methods involve training Word2Vec with individual words (*i*.*e*. the sentences are split on whitespace or punctuation characters), we used shallow parsing and custom grammar patterns to chunk the sentences in such a way that noun phrases of optimal length were preserved in the training dataset. In addition, we normalized the mentions of genetic strains, which reduced the impact of synonyms and further improved the robustness of the model.

#### Classifiers trained on assay descriptions can predict ATC codes of involved drugs with high accuracy

Although most assays in ChEMBL involve novel molecules which have not even been accepted for clinical studies, approved drugs are often tested alongside new structures—as positive controls. In this work, we annotated the *in vivo* assays in ChEMBL with the ATC codes of approved drugs they tested. We used these codes in visualizations of semantic space (Fig 7) and to evaluate the functional relevance of the observed clusters. In addition, we built four multi-class random forest models predicting ATC codes based on the features extracted from assay summaries. In all classification tasks we considered, the models were able to predict ATC codes with high overall accuracy. We conclude that the descriptions of *in vivo* assays are well-suited for the prediction of ATC codes since they include many anatomical terms as well as phrases describing medicinal effects and therapeutically relevant phenotypes. In addition, the results further confirm that neural embeddings learned by Word2Vec capture true functional similarities between assays.

Importantly, the same classification model can be used to predict the function of novel compounds that have not yet been assigned ATC codes (and by inference a disease/therapeutic area assignment). For ChEMBL users, this would mean easier access to 80% of the *in vivo* assays that do not include approved drugs. In this regard, our method would probably work best as a part of an integrative approach incorporating chemical structure and target information [42] in addition to the features extracted from text. An obvious application of these models is to apply them over non-ChEMBL derived full-text, to suggest a disease association and support hypothesis generation.

#### Information extracted from assay descriptions provides insights into trends in animal model usage in drug discovery

Part of this work involved extraction of animal disease model mentions from text. Due to the lack of annotated training sets and controlled vocabularies this turned out to be a substantial task. Despite the current limitations, we could identify a variety of spontaneous and experimental animal models in the assay descriptions. Since ChEMBL data are derived from scientific literature of more than four decades, we hoped that the results would help us better understand the role of different animal models in preclinical drug discovery. In this section, we discuss some of these findings.

Frequency analysis of extracted terms reveals that mouse and rat strains most commonly mentioned in the ChEMBL assay descriptions include many long-established traditional rodent lines. Amongst the three most frequent models, two strains (Sprague Dawley rat and Swiss mouse) were already established by the 1920s, whilst the Wistar rat, introduced as early as 1906, is the oldest rat strain in biomedical research [43–45]. In addition, as highlighted in Fig 5, the most frequent rodent lines include many traditional outbred strains (*i*.*e*. stocks). These genetically heterogeneous colonies of animals are commonly used in pharmacology as general purpose models. Other researchers reported similar trends, often discussing the disadvantages of using outbred animals with ill-defined genetic background including their negative impact on the *intra*-lab reproducibility of research [44, 46, 47].

In the case of induced disease models, the condition of interest can be experimentally produced in animals of different genetic backgrounds and, indeed, of different species. Here, we analyzed the differential use of rats and mice in various experiments (Fig 10A) as well as across a range of disease areas (Fig 10B). The observed variations may be attributed to several factors. Firstly, mice and rats differ in anatomy. The larger physical size of rats facilitates physiological measurements and surgical procedures. Hence, rats are more often used in assays involving surgery such as ligation of the spinal nerve or bile duct cannulation. Smaller size of mice, on the other hand, is preferred in the tail suspension test—a behavioral assay commonly used to evaluate antidepressant drug candidates. In this experiment, a rodent is suspended by its tail and observed for the extent of active (escape) movement versus passive immobility thought to be characteristic of a depressive-like state [48]. Since rats are too heavy to support their weight by tails only, this test is performed primarily in mice [48]. Secondly, there are known behavioral differences between the two species. Rats are generally considered to be more intelligent and more behaviorally complex and, hence, more useful for some behavioral assays for CNS indications such as elevated plus maze or novel object recognition tasks [49]. Thirdly, some fields are dominated by one species due to availability of well-established models as is the case with mouse xenograft models for cancer research. Finally, the mouse might generally be preferred due to the lower cost associated with caging and maintenance.

### Further applications of this approach

For ChEMBL users, it is difficult to identify which *in vivo* assays correspond to a disease of interest. Some assays involve approved drugs with known indications, however these correspond to just 20% of the dataset. For the remaining 80% assays involving only novel, unannotated structures, there are not many options beyond a simple keyword search, which does not benefit from synonym mapping and in addition requires knowledge of animal models and phenotypes.

Here, we have shown that natural language processing (NLP) and neural language models can be used to automatically classify animal-based assays in ChEMBL based on the information encoded in the free text of assay descriptions. The approach could be further extended by incorporating models based on chemical similarity of associated molecules to known drugs or clinical candidates. Meaningful classification of animal-based assays would provide better access to the currently less widely analyzed *in vivo* screening data in ChEMBL by helping users find a subset of data that are most likely to be relevant.

Apart from application in the use of the ChEMBL resource, our work has applications in translational bioinformatics and data-driven drug discovery [50]. We demonstrated the first attempt to extract the mentions of animal models from text and produced a network of relationships between animal models and approved drugs. Given the functional relevance of *in vivo* screening and substantial differences between different model systems, we believe that bulk analysis of animal-based drug screening data could improve our understanding of biological activities of small molecules as well as the challenges of successful translation. In this regard, text mining techniques will play an important role in extracting and integrating animal model information since most relevant studies are disseminated in the unstructured format in scientific publications and patents.

### Limitations of the approach and future work

Some of the limitations of this work arise from the shortcomings of the NLP workflow. Except for genetic strains, we do not normalize synonyms, although Word2Vec helped identify many related phrases for commonly used expressions (e.g. the metrazole—pentyneletrazole example discussed above). Furthermore, our dictionary-based NER methods favor precision, but may overlook terms that are not covered by the underlying vocabulary. On the other hand, the rule-based method used to identify mentions of experimental animal models include extraction patterns that are highly dataset-dependent and would have to be evaluated and potentially re-optimized before application to documents outside of the ChEMBL corpus.

Other limitations are due to the features of the underlying dataset, including its relatively limited size—larger corpus would certainly improve the performance of computational models we used. In addition, some therapeutic areas are underrepresented resulting in unbalanced training sets. Furthermore, descriptions frequently lack essential information about animal models, and some assays are currently misclassified.

In addition, some of our basic assumptions may be incorrect. For instance, our animal model—approved drug relationships are based on a co-occurrence assumption which might not hold in all cases since approved drugs might not always be active in disease models in which they were tested. Hence, we might have erroneously annotated some assays with incorrect ATC codes.

### Summary

Testing drug candidate molecules in proven, or putative, animal models of mechanistic efficacy is an important part of all drug discovery programmes. Yet, there are almost no publicly available resources storing the results of historical *in vivo* compound screening. A notable exception is ChEMBL—a bioactivity database widely known, primarily for its ligand-protein binding data.

In this work, we demonstrate that *in vivo* assay data in ChEMBL are, despite their largely unstructured format, a valuable resource for direct use in data-driven drug discovery and optimization. We show that the descriptions of screening assays can be effectively and efficiently mined and classified using a combination of modern text processing techniques and neural language models, and that the extracted information, particularly when combined with the structured database content, provides fundamental insights into the inter-relationships of experimental models, drugs, and disease phenotypes. Finally, there is currently an active debate in the literature over the reproducibility of in vivo bioassay results and publication bias in reports of animal studies in general [51], approaches developed in our work have potential to further inform this debate.

## Materials and methods

The goal of this study was to mine the descriptions of animal-based bioassays for information about animal disease models and their role in drug discovery. To this end, we built a system that uses NLP techniques and machine learning models to extract relevant information from the ChEMBL *in vivo* bioassay description dataset and automatically organize extracted concepts as well as whole assay descriptions based on their semantic similarity.

**Fig 11:**
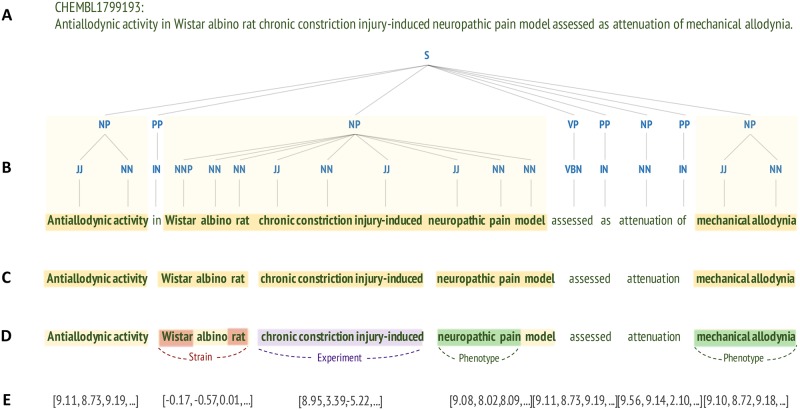
Processing of assay descriptions, with an illustrative example case. **(A)** The input data: raw assay descriptions retrieved from the ChEMBL database. **(B)** Shallow grammatical analysis (shallow parsing). GENIA tagger annotates each word with its corresponding part-of-speech (POS) category (e.g. noun, adjective, verb). The POS annotations are then used to find longer chunks of text corresponding to noun phrases; here represented as yellow blocks in the shallow parse tree. **(C)** Custom chunking. Noun phrases detected by GENIA are simplified using custom tags and chunking rules. **(D)** Named entity recognition (NER). Strains, experimental animal models, and phenotypic terms are identified in terms using a combination of dictionary and rule-based NER methods. **(E)** Learning distributed vector representations. The entire dataset of preprocessed assay descriptions is used to train a neural network language model, Word2Vec. Thus, words and noun phrases from each assay description are converted to high-dimensional numerical vectors that can be used as input for clustering and machine learning models. *S*, sentence; *NP*, noun phrase; *PP*, prepositional phrase; *VP*, verb phrase; *JJ*, adjective; *NN*, noun; *IN*, preposition; *NNP*, proper noun; *VBN*, verb, past participle.

We begin with preprocessing and grammatical analysis of the assay descriptions extracted from ChEMBL (the overview of this step is illustrated by Fig 11). We use the GENIA tagger to tokenize the sentences and to annotate the words with part-of-speech (POS) tags and other linguistic features. Next, we use custom grammatical patterns to chunk the descriptions such that noun phrases of optimal length and content are retrieved. To identify mentions of animal models and their phenotypes in text, we use a combination of dictionary and rule-based named entity recognition (NER) methods that consider lexical and syntactic patterns. Next, we build an unsupervised neural network model to convert text of the descriptions into numerical vectors, which we then use to cluster extracted concepts and to train random forest classifiers. Finally, we combine information extracted from text with curated fields in ChEMBL to associate animal models with approved drugs tested in them.

### Dataset

The first input for our system is a text corpus of experimental descriptions of *in vivo* assays involving animal models of human disease and physiology. To generate the corpus, we identified a subset of relevant experiments in ChEMBL (release 21 [52]) by selecting all functional whole organism-based assays in mouse and rat. There are 100,250 such assays in the database, each summarized by a concise description and a set of curated annotations. The latter include: species name, structures and ATC classification codes of compounds tested in the assay, and details of the publication in which the assay was reported.

To easily query and manipulate the dataset, we indexed the textual descriptions of selected assays and associated structured data with Elasticsearch—a distributed full-text search engine built on Apache Lucene [53]. All SQL queries used to retrieve the data and to calculate their statistics can be found in S2 Text; the data are available in S1 and S2 Datasets.

### Preprocessing and noun phrase extraction

In the text of assay descriptions, complex multi-word noun phrases (NPs) are often used to represent meaningful concepts such as experimental stimuli (*e*.*g*. “high fat diet”), experimental readouts (“systolic blood pressure”, “insulin level”) or assay names (“tail flick assay”, “tail suspension test”). In this work, we use such phrases in the rule-based named entity recognition and as input for the language model. In order to extract NPs from text, we applied shallow parsing analysis (chunking [54]) with custom grammatical patterns. First, we assign each word in a preprocessed description its corresponding part-of-speech (POS) category (e.g. noun, adjective, verb). Next, we search for chunks of text corresponding to individual noun phrases using custom grammatical and lexical rules.

Prior to the syntactic analysis, we modified all assay descriptions as follows: we first normalized the species names, e.g. by substituting each mention of “*Rattus norvegicus*” with “rat”; next, we expanded commonly used acronyms of administration routes (*e*.*g*. sc—subcutaneous(ly)) and administration regimes (*e*.*g*. qd—daily) using a custom dictionary covering 20 common abbreviations (S3 Dataset).

#### POS tagging

Following preprocessing, we used the GENIA tagger (version 3.0.2, February 2016) [55, 56] to tokenize and annotate the descriptions with shallow linguistic features such as POS and chunk tags. GENIA is tuned to the analysis of English biomedical text [55] and in addition to syntactic tagging, it also has biological NER functionality: it can identify and annotate words and phrases corresponding to biological entities including proteins, cell lines, and cell types. An example output of this process is shown in S1 Text (1.1).

#### Chunking and noun phrase extraction

The shallow parsing (chunking) functionality of GENIA makes it easy to extract noun phrases from the annotated sentences. However, the NP chunks produced by the tagger often correspond to complex phrases that might include multiple simple (base) NPs [57] as well as non-informative words such as determiners, *e*.*g*. articles “a” or “the”, or possessive pronouns (“its”) [58]. Thus, noun phrases extracted directly from the GENIA chunking output are often very long and specific: for example, “a perorally dosed Freund's complete adjuvant induced rat inflammatory pain model” is a single such noun phrase.

To achieve a more granular output, we attempted to extract only base noun phrases from descriptions (*e*.*g*. “inflammatory pain model” in the example above). To this end, we combined POS tags assigned by GENIA with custom tags and chunking grammar defined based on our observations of patterns commonly used in assay descriptions. First, we did not allow for determiners, numbers, and other non-informative word types to be part of a noun phrase. These were not excluded only if GENIA tagged them as a part of protein or cell line name. For instance, “***angiotensin 2*** induced blood pressure” and “***Concanavalin A*** induced hepatic cell necrosis” are both allowed in our system. In addition, we defined custom chunking rules based on our observations of patterns commonly used in assay descriptions. For instance, the two example expressions above represent a common type of phrases that combine an experimental stimulus (e.g. “concanavalin A”) and a phenotypic outcome (“hepatic cell necrosis”) linked by the word “induced”. However, in different descriptions the same stimulus might appear with a different phenotypic readout: “concanavalin A induced ALT elevation”, “concanavalin A induced T-cell proliferation”, “concanavalin A induced IL-2 production”, *etc*. To separate these two types of information (stimulus and outcome), we defined a custom chunking rule that splits noun phrases on the word “induced”.

Specifically, we assigned custom tags *IND* and *IND_B* to mark twenty-four keywords related to experimental procedures: “induced”, “infected”, “xenografted”, “fed”, “operated”, “(pre)treated”, “stimulated”, *etc*. (see: S1 Text for the whole list). Similarly, custom tags *TRANSG*, *EXPR*, and *KNOCK* were used for keywords associated with genetically modified animals: “transgenic”, “overexpressing”, “knockout”, *etc*. (S1 Text). Finally, we annotated words corresponding to species names (“rat”, “mouse”) with a custom tag *SP*, while custom tags *PROT* and CELL were used to annotated words that form a part of a protein/cell line name (based on the GENIA NER tags). To extract noun phrases, we then used the RegexpParser function from the Python’s nltk module [59] to search and tag all sequences of words that followed the pattern below:

NP:{<JJ|NN.*>*<PROT|CELL|NN.*>+},

where *NP*, noun phrase; *NN*, noun; *JJ*, adjective; *VBD*, verb (past tense); *VBN*, verb (past participle); *PROT*, protein (custom tag); *CELL*, cell line (custom tag). See S1 Text for additional explanation and examples.

### Named entity recognition (NER)

We adopted two distinct procedures for the detection of relevant concepts in the text of assay descriptions. 1) To identify the names of genetic strains and phenotypes, we used a dictionary-based approach in which documents are matched against comprehensive lists of terms from existing ontologies and custom-built dictionaries. 2) For the detection of induced (experimental) disease models, we adopted a rule-based approach combining syntactic information and custom lexical extraction patterns. We describe the basic aspects of our NER workflow in the section below, while additional details and explanations can be found in S1 Text.

#### Genetic strains

To identify mouse and rat strain mentions in text, we created two dictionaries based on official strain listings and nomenclature guidelines maintained by repositories where new strains are registered [25, 26]. Both dictionaries list basic strains (1,307 mouse and 648 rat strains), together with the strain type (*e*.*g*. inbred or hybrid), available synonyms, and substrains; see S4 and S5 Dataset files.

Our main source for the mouse strain dictionary was the list of strains registered through Mouse Genome Database (MGD [29]). For each inbred strain we gathered all the derived substrains, whilst the names of hybrid, co-isogenic and congenic strains remained as individual terms. To map substrains to their corresponding ancestors, we parsed the names following standard nomenclature guidelines [27, 28]. Specifically, names of substrains are constructed by appending parental strains with the laboratory code(s). For example, AKR/NCr is a substrain derived at Charles River (Cr) laboratory from the NIH (N) substrain of AKR mouse. For each strain and substrain we then gathered additional synonyms from the Nonstandard Mouse Strain and Stock Nomenclature list available from the ftp site of MGD [60]. Examples included in the list are expansions of acronyms that form strain names (“non obese diabetic” for NOD mouse) as well as commonly used strain names that do not follow the official nomenclature (“c57Black” and “c57/black” for the C57BL mouse strain). Finally, we have augmented the dictionary with synonyms from Charles River—a specialist laboratory animal supply website [61], and supplemented with common outbred stocks from a review paper by Chia *et al* [44].

As the main source for the rat strain dictionary we used the Rat Strain Ontology (RSO) [62]–structured vocabulary developed at the Rat Genome Database (RGD [30]). RSO represents registered rat strains in a hierarchical format that allows for easy mapping between derived and parental strains. We used the second level of the RSO hierarchy as the basis for our dictionary (the first level corresponds to the strain type, such as “inbred”, “outbred” or “hybrid”). For each second level term, we collected all child nodes (*i*.*e*. all derived strains) and available synonyms. In addition, we augmented the dictionary with the rat strain list from the MGD website [63] along with strains and synonyms from the Charles River website [61].

We then used the two dictionaries to identify the mentions of rodent strains in the assay descriptions. Upon recognition, we normalized synonyms to preferred names and derived substrains—to the parental strains. To minimize the impact of punctuation variation, we applied a string matching method that only depends on alpha-numerical characters: for example, “ob-ob mouse” is mapped to “ob/ob mouse”. The mentions of strains in the text might also include words such as “female”, “male” or “adult”; for instance, the method detects the mentions of “C57black mouse”, “C56BL/6J mouse”, “C56BL/6J male mouse”, and “Black 6 mouse”, and substitutes them with “C57BL mouse”–the official name for the strain.

#### Phenotypes

Unlike animal model names, phenotypic terms (particularly diseases) are often considered in text-mining analyses of biomedical text. Hence, there exist many terminological resources that can readily be used in dictionary-based NER. In this current study, we selected ten commonly used ontologies to capture phenotype-related terms in assay descriptions. The selected resources are themselves partially overlapping and cover:

- symptoms: Symptom Ontology (SYMP [64]), Clinical Signs and Symptoms Ontology (CSSO [65]),
- human and animal phenotypic traits: Human Phenotype Ontology (HPO [66]), Mammalian Phenotype Ontology (MP [67]), Vertebrate Trait Ontology (VTO [68]), Animal Trait Ontology for Livestock (ATOL [69]),
- clinical measurements: Clinical Measurement Ontology (CMO [70]),
- diseases: Human Disease Ontology (DOID [71]), Mouse Pathology Ontology (MPATH [72]),
- behavioral processes and phenotypes: Neuro Behavior Ontology (NBO [73]).

#### Induced and transgenic animal models

No controlled vocabularies currently exist for induced and transgenic animal models. Here, we applied a rule-based method that identifies relevant terms using manually defined extraction rules. To design these rules, we carefully analyzed a subset of assay descriptions to find textual expressions that represent experimental animals. We then generalized these expressions to a set of eight extraction patterns exploiting lexical and syntactic constraints. For instance, phrases such as “glucose tolerance test”, “Freund's complete adjuvant induced” or “*Staphylococcus aureus* infected” can be captured with the following pattern:

{<NP|FW|POS>*<IND>+},

where NP, noun phrase; FW, foreign word; POS, possessive ending; IND, custom tag (described in the previous section). The extraction patterns and example phrases they capture are fully listed in S1 Text.

#### NER performance analysis

To assess the performance of our NER workflow, we asked two curators to manually annotate 500 randomly selected assay descriptions with four distinct classes of concepts: “genetic strain”, “experimental model”, “transgenic model”, and “phenotype”. The last category includes diseases (*e*.*g*. “influenza”), symptoms (“edema”), signs (“blood pressure”), and behaviors (“writhing”, “stereotypy”), but excludes molecular biomarkers such as “DOPA levels” or “IL4 production”. The annotated sentences (in BRAT format [74]) can be found in S1 File. We calculated the overall NER performance with the standard performance metrics (precision, recall, F1-score) based on exact and partial matches. In addition, we assessed the interannotator agreement (IAA) using the strict and relaxed IAA measures described in [75]. For details and comparison with other methods (*e*.*g*. phenotype annotation with MetaMap), see S1 Text.

### Construction and visualization of semantic space with Word2Vec

In the next step, we converted words and phrases from assay descriptions into numerical vectors that can be used to find semantic similarities between concepts and to train classification models.

To calculate distributed vector representations for the assay description dataset, we used an unsupervised neural network model, Word2Vec [33], implemented in the Python gensim module [76]. As input for the model, we used preprocessed assay descriptions tokenized into words and noun phrases (see: “Chunking and noun phrase extraction” section). For better results, we normalized the names of genetic strains mentioned in the descriptions, and removed numbers and standard English stop words (“the”, “in”, *etc*.) from the training sentences. The parameters of the model were set to standard values as follows: window (the maximum distance between the current and predicted word) = 5; minimum count (minimum word frequency) = 30; number of features (the dimensionality of resulting word embeddings) = 250. The output of the model was a set of 250-dimensional numerical vectors, each corresponding to a single word or phrase from the input corpus.

To find semantic similarity values for pairs of terms, we calculated the cosine distances between their corresponding vector embeddings [33]. We used analogous method to find similarities between entire assay descriptions. In this case, we first converted assay descriptions into numerical format by averaging their corresponding word vectors and normalizing the resulting mean representation vectors to unit norm [77, 78].

We visualized pairwise semantic similarities for a set of 35 most frequent animal models (with an exception of general purpose strains) and 35 phenotypic terms using a hierarchically clustered heatmap implemented in Python seaborn module for statistical data visualization [79].

To qualitatively analyze the distribution of functional assays, we projected the neural embeddings from 250-dimensional space into 2D using t-Distributed Stochastic Neighbor Embedding (t-SNE) [37]. To reduce the noise and speed up the computationally expensive t-SNE calculations, we first reduced the dimensionality of the vectors to 20 dimensions [37] using principal component analysis (PCA) [80].

### Random forest classifiers

Next, we considered the problem of assay classification. To train supervised models, we used the fact that many assays include reference molecules—mostly approved drugs with known indications. Specifically, we used the Anatomical Therapeutic Chemical (ATC) classification codes assigned to those drugs to annotate the assays and divide them into classes [23]. In most cases, we used the second level of the ATC hierarchy corresponding to the main therapeutic group of a drug, *e*.*g*. drugs used in diabetes (“A10”) or in epilepsy (“N03”). We considered different classification problems and built four distinct random forest models that predict assay class membership based solely on the textual information from the descriptions.

The first classifier predicts whether an assay involves any cidal/cytotoxic drugs. These are therapeutics whose main mechanism often involves causing cell death or inhibiting the growth of microbes): antineoplastic drugs (ATC code “L01”), antibacterials (“J01”), antivirals (“J05”), antiprotozoals (“P01”), *etc*. (see S1 Text).

The second model predicts whether an assay involves any drugs acting on the nervous system (ATC code “N”).

The third model is a multiclass classifier that assigns an assay with one of the five most common ATC code (level 2) combinations—a proxy for the most common disease areas in ChEMBL. In order of frequency these are: antiepileptic drugs (ATC code “N03”), psycholeptic drugs (“N05”), antineoplastic drugs (“L01”), drugs used in diabetes (“A10”), and anti-inflammatory drugs (combination of 4 ATC codes: “C01”, “M01”, “M02”, “S01”). The last combination of ATC codes is assigned to a nonsteroidal anti-inflammatory drug, Indomethacin, which is very commonly used as reference standard in the models of inflammation and pain.

The fourth classifier predicts specific drug classes for assays involving drugs acting on nervous system. The six class labels are: antiepileptics (“N03”), psycholeptics (“N05”), analgesics (“N02”), psychoanaleptics (“N06”), antiparkinsonians (“N04”), and anaesthetics (“N01”).

Dividing assays into classes is not a straightforward task since some assays might involve multiple drugs and, in addition, one drug might be assigned multiple ATC codes. To deal with this complexity, we selected a subset of assays that can be unambiguously assigned to one of the classes in every classification problem. For the details on class assignment and number of assays allocated to different classes, see S1 Text.

We applied two different methods for splitting the data into ten subsets used for training and testing in the 10-fold cross-validation procedure. In the first method, we split all assays randomly into equally sized subsets; in the second method, we partitioned the assays by randomly splitting the documents (scientific publications) from which the assay data were curated. This is important since the descriptions of assays reported in the same document are often very similar; commonly, they correspond to the same experiments that differ solely in dose or timing details. The second splitting method assured that such assays would never be used for both model training and testing, which might result in classifiers with overly optimistic performance and poor generalization to new data. See [38] for in-depth discussion on splitting assay data for QSAR model building.

We trained each random forest (RF) model with a set of vectors calculated for assay descriptions as average of their corresponding word embeddings (see previous section). Each RF classifier consists of an ensemble of decision tree estimators trained on an equally sized dataset sample drawn with replacement. The final classification is given by averaging probabilistic predictions of individual decision trees. We set the number of tree estimators to 200; we used adjusted class weights to reduce the impact of dataset imbalance (class_weight parameter set to “balanced”). Other parameters remained set to default values of the Python scikit-learn implementation [81]. Following 10-fold cross-validation procedure, we calculated standard performance measures (precision, recall, accuracy, F1-score), confusion matrix, and out-of-bag estimate (OOB) [82] for each model. Additional details on performance and comparison with other text-based methods (paragraph2vec and bag-of-words with TF-IDF weighting) are reported in S1 Text.

### Network of drugs and animal models

To generate a network of drugs and animal models, we combined the information extracted from assay descriptions with curated compound data from ChEMBL. For a subset of assays involving approved drugs, we created a dataset of animal disease models, including experimental and transgenic models as well as genetic strains. From the latter, we excluded general purpose models (such as Wistar rat or Swiss mouse), which are indiscriminately used for screening compounds for diverse indications (leaving only strains that spontaneously develop disease-related phenotypes). In addition, we manually consolidated synonymous names of experimental disease models; for instance “maximal electroshock”-“maximum electric shock”, “pentylenetetrazole (PTZ)”-“pentylenetetrazole”, and “hot plate”-“hotplate” are pairs of equivalent terms which were merged together. To create the network, we linked animal models to approved drugs such that a drug is connected to an animal model if it was tested in at least five assays involving this model. This resulted in a bipartite graph with 554 nodes and 710 edges, which we visualized using the Gephi network visualization software [83].

## Supporting information

S1 Text Additional explanation of methodology, together with supplementary data tables and figures. (DOCX)

S2 Text SQL queries used to retrieve data from ChEMBL. (PDF)

S1 Dataset *In vivo* bioassay data used in the analysis. Dataset includes descriptions and curated annotations of *in vivo* bioassays as well as information about associated scientific articles.
(XLSX)

S2 Dataset Compound data. Dataset includes names, identifiers, and ATC codes of compounds tested in individual *in vivo* bioassays.
(XLSX)

S3 Dataset Administration acronyms. List of administration acronyms, together with definitions.
(XLSX)

S4 Dataset Mouse strain dictionary. List of mouse strains and stocks, together with associated substrains and synonyms.
(XLSX)

S5 Dataset Rat strain dictionary. List of rat strains and stocks, together with associated substrains and synonyms.
(XLSX)

S6 Dataset List of generated animal model—Drug relationships. List of relationships used to generate the animal model—drug network.
(XLSX)

S1 File Manual annotations of 500 randomly selected assay descriptions. Manually annotated assay descriptions (consensus corpus) in BRAT format.
(ZIP)
