# Supplementary material for: Classification and analysis of a large collection of in vivo bioassay descriptions
Source: PLoS Comput Biol. 2017 Jul 5;13(7):e1005641. doi: 10.1371/journal.pcbi.1005641 (PMC5517062; doi:10.1371/journal.pcbi.1005641)
Supplement: S1 Text — (DOCX) [file pcbi.1005641.s001.docx]

**Supplementary information**

1. **Chunking and noun phrase extraction**

**1.1. Output generated by the GENIA tagger for an example assay description (ChEMBL assay id: CHEMBL1028930).**

For each word in the input sentence (first column), GENIA first finds its base form and its part of speech (POS). Based on the POS annotation (third column), it then assigns each word with a chunk tag. Tags B-NP and I-NP, for example, mark the words that form part of a noun phrase: its beginning and continuation, respectively. Finally, the fifth column contains named entity tags assigned to biological entities such as proteins or cell lines.

As shown by figure below, there are four noun phrases in the example assay description: “increase”, “insulin levels”, “human BT474 cells”, and “SCID mouse”. In addition, the sentence contains two biological entities: “insulin” and “human BT474 cells”.

Acronyms: *NN*, noun; *IN*, preposition; *NNS*, noun, plural; *JJ*, adjective; *VBD*, verb, past tense; *NP*, noun phrase; *PP*, prepositional phrase; *VP*, verb phrase, *B-*, beginning of phrase; *I-*, inside phrase; *O-*, outside phrase.

|  |  |  |  |  |
| --- | --- | --- | --- | --- |
| Word | **Base form** | **Part-of-speech** | **Chunk** | **Named entity** |
| Increase | increase | NN | B-NP | O |
| in | in | IN | B-PP | O |
| insulin | insulin | NN | B-NP | B-protein |
| levels | level | NNS | I-NP | O |
| in | in | IN | B-PP | O |
| human | human | JJ | B-NP | B-cell_line |
| BT474 | BT474 | NN | I-NP | I-cell_line |
| cells | cell | NNS | I-NP | I-cell_line |
| xenografted | xenografte | VBD | B-VP | O |
| SCID | SCID | NN | B-NP | O |
| mouse | mouse | NN | I-NP | O |

**1.2. Custom tags used in chunking and noun phrase extraction**

In addition to the default tags assigned by GENIA, we have further extended the annotation of assay descriptions with eight custom tags, defined in the table below. The tags were then used in combination with custom-defined grammar rules to extract noun phrases (see Fig B) and to identify names of induced and transgenic animal models (see Fig D).

| Custom tag | Keywords | Comment |
| --- | --- | --- |
| SP | mouse, rat | Species names |
| IND | induced, (pre)treated, stimulated, challenged, fed, loaded, fasted, infected, xenografted, injected, operated, ligated, implanted, lesioned, allografted, injured, transplanted, pithed, inoculated;  assay, test, procedure, studies;  challenge, injection(s), infection(s), inoculation, (pre)treatment, ligation, operation, stimulation, xenograft, injury, infusion, overloaded | Experimental treatment keywords and hyphenated phrases that involve them (*e.g.* carrageenan-induced, formalin-challenge) |
| IND_B | bearing | Keyword “bearing”, *e.g.* in “mouse bearing leukemia P388 cells” |
| PROT | *e.g.* PGE2, adenosine A3 receptor, human glucagon receptor | Words recognized as part of a protein name by the GENIA tagger |
| CELL | *e.g.* B16 melanoma, L1210 leukemia cell line, human PC3 cells | Words recognized as part of a cell line name by the GENIA tagger |
| TRANS | transgenic, knockout, knock-out, knock-in | Keywords used in names of transgenic animals, as well as tokens that end with “KO” (*e.g.* H1RKO) and tokens that start with “Tg” (*e.g.* Tg2576) |
| EXPR | expressing, overexpressing, deficient | Keywords used in names of transgenic animals as well as tokens that end with “expressing” (*e.g.* KIAA1263-expressing) or “deficient” (*e.g.* PTEN-deficient) |
| KNOCK | knock | Keyword “knock” and words that end with it (*e.g.* CRTH2-knock) |

**1.3. Noun phrases extracted for the same assay descriptions using GENIA (above) and custom chunking rules (below).**

Noun phrases can be extracted directly from the GENIA output using the assigned chunk tags; see Table A. However, the default noun phrases identified by the tagger are often too long and specific for our application. To achieve a more appropriate, granular output, we searched for shorter noun phrases using a simple chunking grammar.

The grammar consists of two regular-expression rules that indicate how to find a noun phrase in an annotated sentence:

$$NP:\{<JJ|NN.*>*<PROT\left| CELL \right|NN.*>+\}$$

$$NP:\{<SP>\}$$

The upper rule tells the chunker to extract a noun phrase, whenever it finds one or more nouns (NN.*), protein names (PROT) or cell line names (CELL) optionally preceded by any number of adjectives or nouns; the lower rule tells the chunker to extract species names. Importantly, non-informative words such as articles (“a”, “the”) or numbers are not allowed in the grammar.

The figure below shows two parse trees generated for the same assay description. In each tree, the words are tagged and grouped into chunks which together form entire sentence. Noun phrases are highlighted in yellow. The upper parse tree was generated using the default GENIA output. There are three noun phrases: “mortality rate”, ‘’Trypanosoma cruzi Y”, and “IFN-gamma knockout mouse Chagas disease model”. In the lower parse tree, this last long noun phrase is broken to shorter constituents using a custom chunking grammar. Custom tags (highlighted in red) are later used in the rule-based NER step to combine noun phrases with appropriate keywords in order to find induced and transgenic animal models: “Trypanosoma cruzi Y *infected*” and “IFN-gamma *knockout*”; see Fig D.

Acronyms: *S*, sentence; *NP*, noun phrase; *IN*, preposition; *VBD*, verb, past tense; *NN*, noun; *NNP*, proper noun.

**1.4. Most common (multiword) noun phrases extracted from assay descriptions.**

Using the method described above, we extracted 21,246 multiword noun phrases from the text of assay descriptions. These were then used as input for machine learning models and for the rule-based NER. The table below shows 20 most common multiword noun phrases extracted with the method.

|  | Noun phrase | Count |  | Noun phrase | Count |
| --- | --- | --- | --- | --- | --- |
| 1 | anticonvulsant activity | 4,840 | 11 | antidiabetic activity | 1,424 |
| 2 | antiinflammatory activity | 4,243 | 12 | life span | 1,138 |
| 3 | antitumor activity | 3,045 | 13 | acetic acid | 1,040 |
| 4 | analgesic activity | 2,652 | 14 | body weight | 924 |
| 5 | mg/kg dose | 2,219 | 15 | percent change | 906 |
| 6 | paw edema | 1,820 | 16 | percent inhibition | 865 |
| 7 | antinociceptive activity | 1,803 | 17 | antiobesity activity | 843 |
| 8 | effective dose | 1,621 | 18 | blood glucose level | 813 |
| 9 | maximal electroshock | 1,549 | 19 | percent increase | 806 |
| 10 | antihypertensive activity | 1,505 | 20 | systolic blood pressure | 801 |

1. **Named entity recognition (NER)**

**2.1. Mapping parental inbred strains to substrains.**

Mouse and rat strains are named according to standardized nomenclature rules that reflect breeding strategy and parental background of the animals. Figure below shows the origin of 10 distinct substrains of inbred CBA mouse. Names of substrains are constructed by appending parental strains with specific laboratory code(s). For example, CBA/CaJ is a substrain derived at the Jackson Laboratory (J) from the Carter (Ca) substrain of CBA mouse. In our mouse strain dictionary (described in the main Methods section), all substrains shown on the figure are mapped to the parental strain – CBA.

Laboratory codes: *Ca*, T.C. Carter; *H*, MRC Mammalian Genetics Unit, Harwell; *N*, National Institutes of Health; *Wehi*, Walter and Eliza Hall Institute of Medical Research; *J*, The Jackson Laboratory; *Anu*, Australian National University; *Narl*, National Laboratory Animal Centre; *Cr*, NCI, DCTD Animal Production Program; *Bom*, M&B A/S.

**
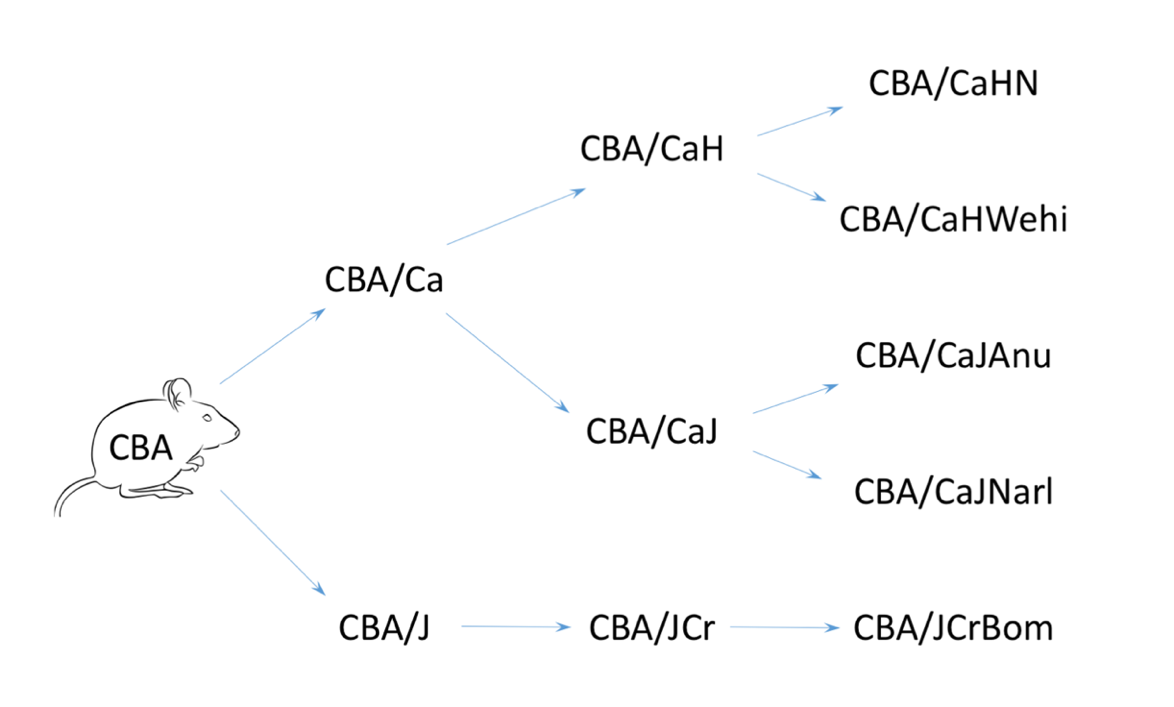
**

**2.2. Most common strains detected in the assay descriptions.**

The table shows twenty most common rodent strains detected in the text of assay descriptions using a dictionary-based approach. Upon recognition, we normalized synonyms to their preferred names and substrains – to parental strains. We carried the dictionary-based NER step before chunking analysis described in the previous section. For the grammatical analysis with GENIA, the detected strain names were temporarily substituted with species names (“mouse” or “rat”) to simplify sentence chunking. We found that this step has improved the quality of the GENIA analysis since many strain names contain short words and numbers that could be assigned incorrect POS tags by the standard GENIA tagger.

|  | Strain | Count |  | Strain | Count |
| --- | --- | --- | --- | --- | --- |
| 1 | Sprague Dawley rat | 6,109 | 11 | db/db mouse | 738 |
| 2 | Wistar rat | 5,013 | 12 | DBA mouse | 688 |
| 3 | Swiss mouse | 2,969 | 13 | Swiss Webster mouse | 501 |
| 4 | C57BL mouse | 2,931 | 14 | Lewis rat | 467 |
| 5 | SHR rat | 1,963 | 15 | CDF1 mouse | 465 |
| 6 | BALB (BALB/c) mouse | 1,680 | 16 | ob/ob mouse | 456 |
| 7 | CD-1 mouse | 1,368 | 17 | Kunming mouse | 386 |
| 8 | CF-1 mouse | 1,288 | 18 | ZDF rat | 381 |
| 9 | ICR mouse | 1,204 | 19 | DDY mouse | 381 |
| 10 | B6D2F1 mouse | 859 | 20 | athymic nude mouse | 370 |

**Grammar patterns used in induced/transgenic rule-based NER and example phrases they capture.**

We used eight custom defined grammatical patterns to capture names of induced and transgenic animal models. Similar to the chunking grammar described in the caption of Fig B, the rules are regular expressions indicating sequences of tags that capture named entities. For example, the first rule: {<IND><IN><NP|FW+>}, corresponds to an experimental treatment keyword such as “induced” or “infected” followed by a preposition (“by”, “with”), followed by a noun phrase or any number of foreign words: “acetic acid”, “Candida albicans”, *etc*. All rules were used with RegexpParser function from the Python’s nltk module for natural language processing [[1](#_ENREF_1)].

1. {<IND><IN><NP|FW+>}

example phrases captured: implanted with P388 leukemia cell line, induced by ethanol, inoculation with L1210 leukemia cell line, injection of acetic acid, infected with influenza A virus, infected with Plasmodium berghei K173, infected with Candida albicans, fed on high fat diet

1. {(<NP|JJ|FW|POS|VBN>*(<NP><\(><NP><\)>)?)*<IND>+ (<\(><NP><\)>)*}

example phrases captured: carrageenan challenge, maximal electroshock induced, glucose tolerance test, hot plate assay, drug discrimination studies, Freund's complete adjuvant induced, maximal electroshock seizure (MES) test, Triton WR-1339 induced, Staphylococcus aureus infected, maximal electric shock assay (MES)

1. {(<NP><IND_B>) | (<IND_B><NP>)}

example phrases captured: bearing P388 leukemia, bearing Ehrlich ascites carcinoma, sarcoma 180 tumor bearing

1. {(<NP>? <EXPR>)? <NP|CD>? <TRANS><CD><SP>}

example phrases captured: transgenic 2576 mouse

1. {(<NP>? <EXPR >)? <NP|CD>? <TRANS><NP>?}

example phrases captured: human PXR expressing transgenic, 1.3.32 transgenic, H1RKO, LDL receptor knockout, human CCR2 knock-in

1. {<SP><EXPR ><NP>}

example phrases captured: mouse expressing human glucagon receptor, mouse expressing human SOD1 G93A mutant, mouse overexpressing PRCP, mouse expressing wild type MCHR1 gene

1. {<NP>? <EXPR><SP>}

example phrases captured: ApoE deficient mouse, CD147-deficient mouse, p38alpha T106M mutant expressing mouse

1. {<NP>? <KNOCK><RP|IN>}

example phrases captured: GPR119 knock out, human D1 receptor knock in, PDE10A-knock out

**2.3. Induced/transgenic model NER.**

The figure below shows how the NER rules are applied to annotated assay descriptions to capture names of induced and transgenic animal models.

Two such names were recognized in the example sentence. “*Trypanosoma cruzi Y infected*” follows {<NP><IND>} pattern (noun phrase followed by the IND custom tag) and, hence, can be captured with the second NER rule described above. Similarly, “*IFN-gamma knockout*” follows the {<NP><TRANS>} pattern captured by the fifth rule.

**Table below** shows twenty most common names of induced/transgenic models extracted from assay descriptions using the proposed approach. We carried the rule-based NER following tagging and chunking of assay descriptions described in the previous section. The extracted phrases were post-processed to remove units (“mg/kg‘’, “hours”, *etc*.) and route of administration keywords (“oral”, “subcutaneous”, *etc*.; see S2 Dataset).

|  | Model | Count |  | Model | Count |
| --- | --- | --- | --- | --- | --- |
| 1 | **Carrageenan induced** | **3,062** | **11** | **Glucose tolerance test** | **444** |
| 2 | **Carrageenan challenge** | **1,150** | **12** | **Hot plate test** | **444** |
| 3 | **Maximal electroshock induced** | **1,048** | **13** | **Scopolamine induced** | **422** |
| 4 | **Acetic acid induced** | **693** | **14** | **Adjuvant induced** | **379** |
| 5 | **Pentylenetetrazole induced** | **594** | **15** | **Streptozotocin induced** | **373** |
| 6 | **LPS induced** | **573** | **16** | **Tail flick test** | **350** |
| 7 | **fasted** | **542** | **17** | **DMBA induced** | **347** |
| 8 | **Diet induced** | **531** | **18** | **LPS challenge** | **321** |
| 9 | **Apomorphine induced** | **465** | **19** | **Acetic acid challenge** | **314** |
| 10 | **Formalin induced** | **465** | **20** | **Implanted with P388 leukemia** | **273** |

To extract the names of experimental stimuli (e.g. “carrageenan” from “carrageenan induced”, “carrageenan challenge”, and “injected with carrageenan”), the phrases can be simply stripped of keywords^[[1]](#footnote-1)^ defined in Table A and prepositions such as “with” and “of”. Table below shows twenty most common stimuli with mention counts across the entire set of assay descriptions.

|  | Model | Count |  | Model | Count |
| --- | --- | --- | --- | --- | --- |
| 1 | **Carrageenan** | **4,613** | **11** | **High fat diet** | **464** |
| 2 | **Maximal electroshock** | **1,103** | **12** | **Streptozotocin** | **390** |
| 3 | **Acetic acid** | **1,058** | **13** | **Scopolamine** | **458** |
| 4 | **LPS** | **1,031** | **14** | **Glucose (e.g. glucose-loaded)** | **350** |
| 5 | **Formalin** | **795** | **15** | **Ovalbumin** | **348** |
| 6 | **Pentylenetetrazole** | **714** | **16** | **DMBA** | **348** |
| 7 | **Apomorphine** | **565** | **17** | **cholesterol** | **282** |
| 8 | **Diet** | **531** | **18** | **Reserpine** | **275** |
| 9 | **Adjuvant** | **471** | **19** | **P388 leukemia** | **273** |
| 10 | **Scopolamine** | **469** | **20** | **Collagen** | **260** |

To further improve the recall, we used the generated list of experimental stimuli to identify mentions of experimental models that could not be covered by the rules described in Text A.

We selected a subset of stimuli with at least 10 mentions in the corpus (537 phrases in total) and used a simple string matching method to tag additional noun phrases in the processed assay descriptions.

As an example, consider the following sentences: (1) “Analgesic activity in Swiss mouse assessed as reaction time after 30 mins by *hot plate test*” and (2) “Analgesic potency in mice after icv injection and 10 min before exposing to *hot plate*”. Using the rules described in Text A, the phrase “hot plate” (experimental device used to evaluate the efficacy of analgesic drugs) could only be detected in the first description as it appears with the keyword “test”. Enhancing the NER with the list of common experimental stimuli allows us to capture the phrase in both descriptions.

1. **NER performance**

**3.1. Examples of manually annotated assay descriptions.**

To evaluate the performance of our NER methods, we asked two curators (a drug discovery scientist and a postgraduate student) to manually annotate a set of 500 randomly selected assay descriptions (0.4% of entire corpus). We asked them to annotate the mentions of animal models belonging to three categories: experimental animal models, genetic strains, transgenic animals, and phenotypes. The first category includes names of experimental interventions used to induce the disease (or phenotype) in the animal, as well as assay names (*e.g.* “forced swimming assay”). The phenotype category includes diseases (*e.g.* “influenza”), symptoms (“edema”), signs (“blood pressure”), behaviors (“writhing”), but excludes molecular biomarkers such as “DOPA levels” or “IL4 production”.

**
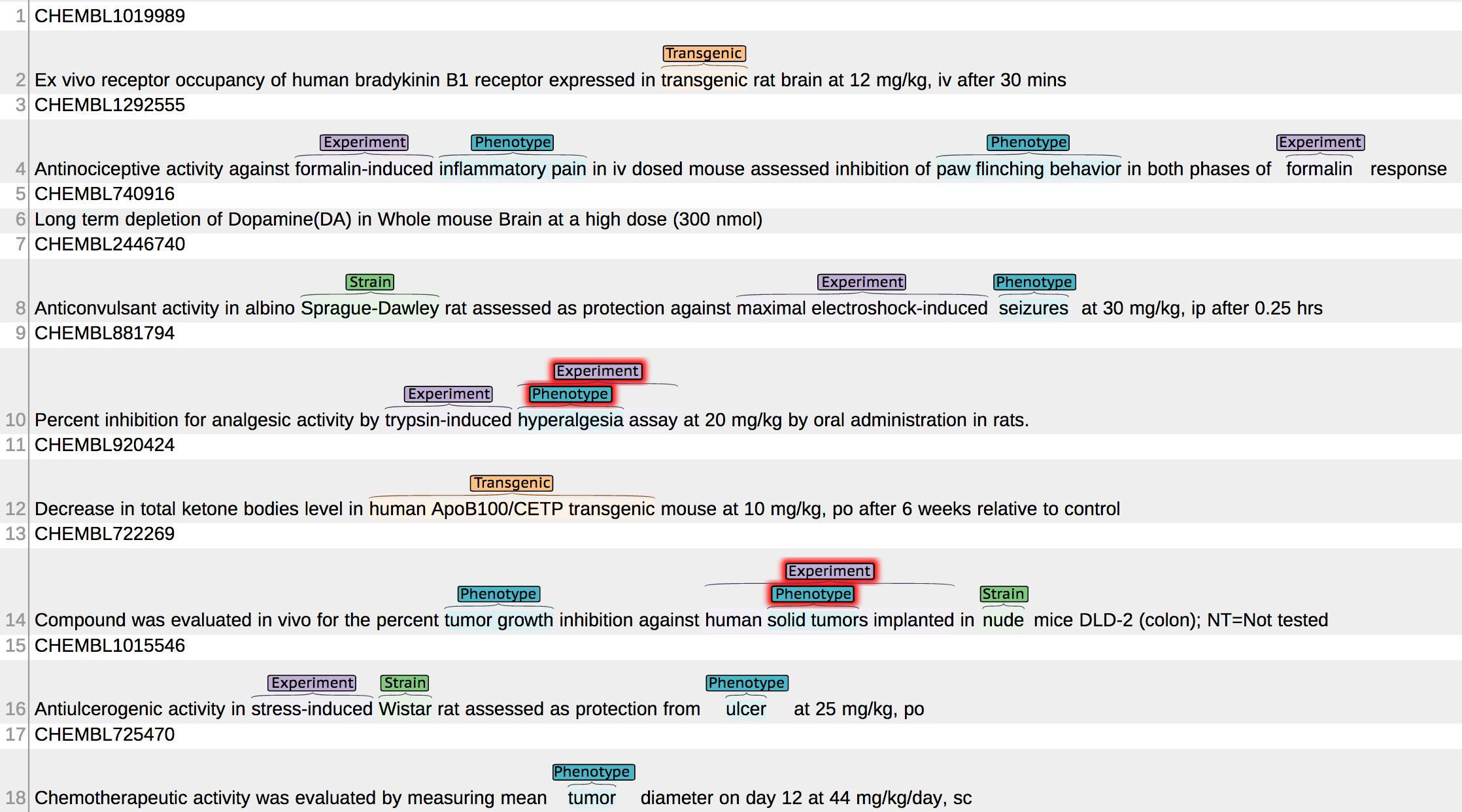
**The figure below shows annotations of ten example assay descriptions as a BRAT viewer snapshot (brat rapid annotation tool [[2](#_ENREF_2)]). The annotations are available in BRAT format in S1 File.

**3.2. Interannotator agreement.**

We measured the inter-annotator agreement (IAA) between the double-annotated assay description set using the strict and relaxed IAA measures described in [[3](#_ENREF_3)]. In general, IAA is measured as fraction of matching annotations between sentences annotated by different curators:

$$IAA= \frac{matches}{matches+non\_matches}$$

In strict IAA, only exact matches are taken into account; in relaxed IAA, partial matches are counted as a half match [[3](#_ENREF_3)]. Partial matches correspond to annotations of partially overlapping spans of text, where annotators agree on the type of entity, but disagree on the exact text boundary. Representative examples of disagreement are listed below:

- “4% sodium chloride-induced” and “sodium chloride-induced” (partial match)
- “Wistar Charles River” and “Wistar” (partial match)
- “compound 48/80 induced lethality test” tagged by one annotator; “compound 48/80 induced” and “lethality test” tagged by the second annotator separately (partial match)
- “pressor response” tagged as Experiment by only one annotator
- “Carworth farms” tagged as Strain by only one annotator
- “DIO” tagged as Experiment by only one annotator
- “habituated” tagged as Phenotype by only one annotator
- “sciatic nerve tibialis muscle contraction” and “muscle contraction”

**3.3. NER performance measures.**

To measure the performance of our NER methods, we used the standard performance measures of precision and recall as well as their harmonic mean – F1 score:

$Precision=\frac{TP}{TP+FP}$ $Recall=\frac{TP}{TP+FN}$

$$F_{1}=2\cdot\frac{Precision \cdot Recall}{Precision+Recall}$$

where *TP* are true positives (hits), *FP* – false positives, and *FN* – false negatives.

Similar to our method for calculating inter-annotator agreement, we used strict and relaxed performance measures. In the first case, only the exact matches contribute to the true positives score (TP); in the latter case, partial matches are accepted as well.

Representative examples of misclassification:

- False negatives:
  - “B6D2F1/jico”, “Jcl:MCH”, “abdominal constriction” (phrases missing from dictionaries)
  - “ovariectomized”, “electrically stimulated”, “B16F10 melanoma” (not covered by the NER rules and the generated list of experimental stimuli)
- False positives:
  - “RNase protection assay” tagged as experimental model
  - “shock” tagged as phenotype in “maximal electric shock assay”
- Partial matches:
  - “shaking” vs “wet-dog shaking”, “extrasystole” vs “ventricular extrasystole”
  - “normotensive pithed” vs “pithed”, “acute colitis” vs “colitis”
  - “ependymoblastoma”, “tumor” vs “ependymoblastoma tumor”

**3.4. Performance of the NER approach**

The table shows precision, recall, and F-score performance measures calculated based on exact and partial matches for four NER tasks: detection of genetic strains, induced (experimental) models, transgenic animals, and phenotypes. The “Count” column shows the number of annotations and fraction of assays involving at least one annotation of each type in the consensus dataset of 500 manually annotated assay descriptions.

| Task | Method | Count | Assay count | Precision | Recall | F1 score |
| --- | --- | --- | --- | --- | --- | --- |
| Genetic strains | exact | 183 | 183 (37%) | 0.994 | 0.913 | 0.952 |
|  | partial | 183 | 183 (37%) | 0.994 | 0.929 | 0.960 |
| Induced models | exact | 389 | 319 (63%) | 0.848 | 0.792 | 0.819 |
|  | partial | 389 | 319 (63%) | 0.885 | 0.833 | 0.858 |
| Transgenic models | exact | 13 | 13 (3%) | 1.0 | 1.0 | 1.0 |
|  | partial | 13 | 13 (3%) | 1.0 | 1.0 | 1.0 |
| Phenotypes | exact | 362 | 282 (56%) | 0.613 | 0.601 | 0.607 |
|  | partial | 362 | 282 (56%) | 0.756 | 0.729 | 0.742 |

**3.5. Comparison with other methods**

To further evaluate our approach, we compared it to the performance of existing methods (where available) on the set of 500 manually annotated assay descriptions.

**Genetic strain detection.** We first compared our strain detection method to structured strain annotations included in the ChEMBL database (assay_strain field). The results are shown in the table below:

| Approach | Method | Precision | Recall | F1 score |
| --- | --- | --- | --- | --- |
| Proposed method | exact | 0.994 | 0.913 | **0.952** |
|  | partial | 0.994 | 0.929 | **0.960** |
| ChEMBL annotations | exact | 0.742 | 0.601 | 0.664 |
|  | partial | 0.928 | 0.753 | 0.831 |

Unlike the assay descriptions, strain annotations in ChEMBL were originally not manually curated but automatically retrieved using a regexp-based method and a basic dictionary [personal communication]. Our method shows higher performance since it is based on a large comprehensive dictionaries generated from official listings of all registered mouse and rat strains. One reason for lower precision of the ChEMBL annotations is that they tag the word “albino” as strain name, which is incorrect (in fact, many distinct strains used in drug discovery, and biomedical research in general, are albinotic). Lower recall observed for ChEMBL annotations is due to a large number of overlooked strain mentions.

**Phenotype detection.** Our proposed method for phenotype detection is based on dictionary-based NER with terms from 10 selected ontologies: VT, MP, SYMP, HP, ATOL, MPATH, DOID, CSSO, CMO, and NBO. We compared our Python implementation with three existing approaches:

- NCBO annotator [[4](#_ENREF_4)] – a dictionary-based customizable NER tool for mapping concepts from biomedical text to UMLS thesaurus and BioPortal ontologies; maintained by The National Center for Biomedical Ontology. We used the web services provided by the NCBO with the following settings: vocabularies restricted to the set of 10 ontologies listed above, minimum match length set to 4 characters.
- MetaMap [[5](#_ENREF_5)] – a highly-customizable tool for mapping concepts from biomedical text to UMLS thesaurus based on symbolic, natural-language processing (NLP) and computational-linguistic techniques; maintained by National Library of Medicine. We used MetaMap in two modes:
  - default settings: allowing for annotations of all semantic types with concepts from the entire UMLS metathesaurus
  - customized settings: restricting the annotations to a small set of relevant vocabularies (MedlinePlus, MeSH, ICD-10, Human Phenotype Ontology, SNOMED, OMIM) and a small set of relevant semantic types (Pathologic function, Physiologic Function, Organ or Tissue Function, Acquired Abnormality, Organism Function, Mental or Behavioral Dysfunction, Neoplastic Process, Clinical Attribute, Finding, Organism Attribute, Daily or Recreational Activity, Sign or Symptom, Individual Behavior, Disease or Syndrome, Behavior, Laboratory or Test Result).
- DNorm [[6](#_ENREF_6)] – a machine learning-based tool for identification and normalization of disease names from biomedical text.

| Approach | Method | Precision | Recall | F1 score |
| --- | --- | --- | --- | --- |
| Proposed method | exact | 0.613 | 0.602 | 0.608 |
|  | partial | 0.755 | 0.731 | 0.743 |
| NCBO Annotator (restricted ontologies) | exact | 0.618 | 0.597 | 0.608 |
|  | partial | 0.759 | 0.723 | 0.741 |
| MetaMap (default settings) | exact | 0.037 | 0.557 | 0.069 |
|  | partial | 0.062 | 0.943 | 0.117 |
| MetaMap (restricted settings) | exact | 0.373 | 0.563 | 0.449 |
|  | partial | 0.511 | 0.760 | 0.611 |
| DNorm | exact | 0.448 | 0.160 | 0.236 |
|  | partial | 0.720 | 0.257 | 0.379 |

The difference in performance between the five methods is largely due to the underlying vocabularies. For our proposed method we have carefully selected a set of 10 ontologies that are suitable for the detection of phenotypic concepts in the text of *in vivo* assay descriptions. This included animal-specific vocabularies such as Mammalian Phenotype Ontology or Mouse Pathology Ontology. For NCBO annotator, we have restricted the concept annotation to the same set of 10 ontologies and the observed performance was comparable with our implementation.

Other methods work with vocabularies that might be less suited for the detection of concepts in the context of animal studies: MetaMap works natively only with human-centered UMLS vocabularies, whilst DNorm is based on the NCBI disease corpus and MEDIC vocabulary, which integrates OMIM and the “Diseases” branch of MeSH.

MetaMap achieves the highest recall (partial matches); however, it’s precision is lower than that of the methods based on the selected set of ontologies. When used with default settings, MetaMap tags concepts of various semantic types (including drugs, units, *etc*.), leading to a very high recall, but very low specificity. Restricting vocabularies and semantic types to a smaller relevant set decreases the recall, but increases precision considerably.

DNorm achieves good precision for partial matches, but it has a very low recall. As a disease- focused resource, it overlooks non-disease terms considered in the analysis (symptoms, *etc.*). In addition, we observed several problems with phrase boundaries detected by DNorm; for instance, “mean tumor” an “inhibit lymphoma” were tagged erroneously, instead of “tumor” and “lymphoma”. This problem contributes to the large difference in precision values observed for partial and exact matches.

1. **Random forest classifiers**

**4.1. Classification of assays involving cytotoxic and non-cytotoxic drugs**

For the first classification problem, we first divided all drugs into two groups: cytotoxic (or cidal) drugs (whose primary functional effect is to act through a mechanism causing cell death, or otherwise inhibiting the growth of microbes); and non-cytotoxic drugs that act through other mechanisms. The first category includes therapeutics used in the treatment of cancer (assigned ATC code “L01”) and infectious diseases including bacterial (“J01”), viral (“J05”), and protozoal (“P01”) infections. To divide assays into classes we assigned “cytotoxic” label to all assays that contain at least one drug with at least one of the “cytotoxic” ATC codes listed in the table below.

| Label | ATC (level 2) codes | Number of assays |
| --- | --- | --- |
| Cytotoxic | - A07 (Intestinal anti-infectives) - D01 (Antifungals for dermatological use) - D06 (Antibiotics and chemotherapeutics for dermatological use) - D08 (Antiseptics and disinfectants drugs) - G01 (Gynecological anti-infectives and antiseptics) - J01 (Antibacterial drugs) - J02 (Antimycotic drugs) - J04 (Antimycobacterials) - J05 (Antiviral drugs) - J06 (Immune sera and immunoglobulins) - J07 (Vaccines) - L01 (Antineoplastic drugs) - L02 (Endocrine therapy) - L03 (Immunostimulants drugs) - L04 (Immunosuppressants drugs) - P01 (Antiprotozoal drugs) - P02 (Anthelmintic drugs) - P03 (Ectoparasiticides) | 3,818 |
| Non-cytotoxic | All other ATC codes | 15,400 |

**4.2. Classification of assays involving drugs acting on nervous system.**

For the second classification problem, we divided all drugs into two groups: drugs acting on the nervous systems and otherwise. The first category includes therapeutics assigned at least one ATC code from the “Nervous system” branch of the ATC classification; the second category includes all the other drugs. To divide assays into classes we assigned “CNS drugs” label to all assays that contain at least one drug acting on the nervous system and “non-CNS” label otherwise.

| Label | ATC (level 1) code | Number of assays |
| --- | --- | --- |
| CNS drugs | N (Nervous system) | 6,830 |
| Non-CNS drugs | All other ATC codes | 12,388 |

**4.3. Classification of assays involving five most common drug classes**

For the third classification problem, we first annotated each assay with all the ATC (level 2) codes across all involved drugs. We then found five most frequent ATC code combinations as a proxy for the most common disease areas represented in the ChEMBL dataset. Next, we chose all assays involving one of the five selected ATC code combinations to train the random forest classifier. The table below shows the selected classes and the number of corresponding assays.

| Label | ATC (level 2) code combination | Number of assays |
| --- | --- | --- |
| Antiepileptics | N03 (Antiepileptic drugs) | 1,857 |
| Psycholeptics | N05 (Psycholeptic drugs) | 1,684 |
| Antineoplastic | L01 (Antineoplastic drugs) | 1,485 |
| Antidiabetics | A10 (Drugs used in diabetes) | 1,175 |
| Anti-inflammatory | C01 (Cardiac therapy); M01 (Anti-inflammatory and antirheumatic drugs); M02 (Topical products for joint and muscular pain); S01 (Ophthalmological drugs). These ATC codes are assigned to a nonsteroidal anti-inflammatory drug, Indomethacin, which is very commonly used as reference standard in the models of inflammation and pain. | 1,024 |

**4.4. Classification of assays involving specific subclasses of drugs acting on nervous system.**

In the fourth classification problem, we built a model predicting the specific subclasses CNS drugs tested in the assays. To train the classifier, we selected assays involving drugs acting on the nervous system (see Text E) that are associated with a single ATC level 2 subclass.

| Label | ATC (level 2) code | Number of assays |
| --- | --- | --- |
| Antiepileptics | N03 (Antiepileptic drugs) | 1,881 |
| Psycholeptics | N05 (Psycholeptics drugs) | 1,736 |
| Analgesics | N02 (Analgesic drugs) | 1,181 |
| Psychoanaleptics | N06 (Psychoanaleptics) | 889 |
| Antiparkinsonians | N04 (Antiparkinsonian drugs) | 387 |
| Anaesthetics | N01 (Anaesthetic drugs) | 252 |

**4.5. Overall performance of the random forest classification models.**

The table below shows the overall performance of the four random forest classifiers calculated through 10-fold cross validation. The performance is reported in terms of mean accuracy and mean out-of-bag (OOB) estimate [[7](#_ENREF_7)]. Accuracy is calculated for the predictions on test set data in each cross-validation round; OOB is calculated for each individual tree in a random forest model based on the data points that were not part of a bootstrap sample used to train the tree (different tree classifiers are trained on different random samples of the dataset).

We applied two different methods for splitting the data into 10 subsets used for training and testing in cross validation. In the first method, we split all assays randomly into equally sized subsets; in the second method, we partitioned the assays by randomly splitting the documents (scientific publications) from which the assay data were curated.

The differences in performance values illustrate the importance of the partitioning method. As shown in the table, the accuracy of predictions was considerably higher for the models built with the first approach. When assay data are split randomly, it is possible that assays curated from the same publication are used for both training and evaluation of the model; since such assays commonly have very similar descriptions, this leads to overly optimistic performance values for the classifier. By contrast, randomly partitioning the dataset based on documents rather than assays assures that such situation does not occur and the final model is more likely to generalize to new data. Analogous observations have previously been made in the context of compound datasets from ChEMBL; see [[8](#_ENREF_8)] for in-depth discussion on splitting assay data for QSAR model building.

In contrast to accuracy, the values of out-of-bag estimate do not differ between the models constructed with different dataset partitioning approaches; in fact, in both cases, the OOB scores are very similar to the “optimistic” accuracy values. This is because OOB estimates are calculated for individual tree classifiers based on data points from the same cross-validation subset.

| Classifier | Accuracy (assay split) | Accuracy  (document split) | Out-of-bag  (assay split) | Out-of-bag  (document split) |
| --- | --- | --- | --- | --- |
| Cytotoxicity | 0.97 | 0.92 | 0.97 | 0.97 |
| CNS vs non-CNS | 0.93 | 0.86 | 0.93 | 0.94 |
| 5 common diseases | 0.94 | 0.87 | 0.94 | 0.95 |
| Nervous system drug subclasses | 0.86 | 0.67 | 0.87 | 0.86 |

**4.6. Comparison with other methods.**

To further evaluate our approach based on averaged Word2Vec embeddings, we compared its accuracy to the performance of two other methods:

- Bag-of-words (BOW) with TF-IDF weightings; implemented in Python scikit-learn library (default settings)
- Paragraph2Vec; implemented in Python gensim module (with the same settings as Word2Vec: window (the maximum distance between the current and predicted word) = 5; minimum count (minimum word frequency) = 30; number of features (the dimensionality of resulting embeddings) = 250)

For each model, vector representations were calculated based on the same input (preprocessed assay descriptions) and used to train the same algorithm (random forest classifier with 200 estimators).

Table below shows the performance of each method reported as mean accuracy in 10-fold cross validation with the document-based dataset partitioning method.

| Classifier | Word2Vec | BOW TF-IDF | Paragraph2Vec |
| --- | --- | --- | --- |
| Cytotoxicity | 0.919 | 0.900 | 0.802 |
| CNS vs non-CNS | 0.863 | 0.843 | 0.711 |
| 5 common diseases | 0.874 | 0.831 | 0.578 |
| Nervous system drug subclasses | 0.673 | 0.658 | 0.511 |

In general, Random Forest models based on averaged Word2Vec embeddings outperform other methods in all four classification problems. However, the accuracy values calculated for the TF-IDF weighted bag-of-words classifiers are only slightly lower, with differences between 0.015 to 0.043; in combination with the ease of implementation, this makes BOW TF-IDF an excellent baseline method for the classification of *in vivo* assay descriptions. Classifiers based on vector representations generated with Paragraph2Vec show the lowest performance, possibly because this model is better suited for large text corpora.

**4.7. Per-class performance of the random forest classifiers.**

To get a better overview of prediction performance of the four random forest models, we calculated per-class performance measures in addition to the overall prediction accuracy. The figures below summarize precision, recall, and F1-score values calculated for individual classes considered in each classification problem. In each case, the classes are ordered by the number of instances (data points) in the test dataset. The same colour palette and scale was used across all figures to enable quick comparison between the classifiers.

The performance values vary considerably between different models. This may be explained by the differences in classification complexity (*e.g.* binary vs multiclass problems) or the dataset size (see Text D - G). In addition, there is a substantial variation in prediction performance between individual classes within a single model. The differences may be explained by the fact that all datasets used to train the models were unbalanced (see Text D – G). Although we used adjusted class weights to reduce the impact of dataset imbalance, lower performance values were typically associated with classes that were underrepresented in the datasets. For instance, in the last classification model there was a three-fold difference in F1-score between the “Antiepileptics” class involving 1,881 instances and the “Anaesthetics” class involving only 252 assays.

**References:**

1. Bird, S., E. Klein, and E. Loper, *Natural language processing with Python*. 2009: " O'Reilly Media, Inc.".

2. Stenetorp, P., et al. *BioNLP shared task 2011: Supporting resources*. in *Proceedings of the BioNLP Shared Task 2011 Workshop*. 2011. Association for Computational Linguistics.

3. Roberts, A., et al. *Semantic annotation of clinical text: The CLEF corpus*. in *Proceedings of the LREC 2008 workshop on building and evaluating resources for biomedical text mining*. 2008.

4. Whetzel, P.L., et al., *BioPortal: enhanced functionality via new Web services from the National Center for Biomedical Ontology to access and use ontologies in software applications.* Nucleic acids research, 2011. **39**(suppl 2): p. W541-W545.

5. Aronson, A.R. *Effective mapping of biomedical text to the UMLS Metathesaurus: the MetaMap program*. in *Proceedings of the AMIA Symposium*. 2001. American Medical Informatics Association.

6. Leaman, R., R. Islamaj Doğan, and Z. Lu, *DNorm: disease name normalization with pairwise learning to rank.* Bioinformatics, 2013. **29**(22): p. 2909-2917.

7. Breiman, L., *Out-of-bag estimation*. 1996, Citeseer.

8. Sheridan, R.P., *Time-split cross-validation as a method for estimating the goodness of prospective prediction.* Journal of chemical information and modeling, 2013. **53**(4): p. 783-790.

1. Here, we used the following subset of keywords: 'induced', 'challenged', 'stimulated', 'injected', 'loaded', 'challenge', 'injection', 'injections', 'treatment', 'pretreatment', 'stimulation', 'implanted', 'infected', 'implantation', 'xenografted', 'xenograft', 'inoculated', 'fed'. [↑](#footnote-ref-1)
