# Supplementary material for: Classification and analysis of a large collection of in vivo bioassay descriptions
Source: PLoS Comput Biol. 2017 Jul 5;13(7):e1005641. doi: 10.1371/journal.pcbi.1005641 (PMC5517062; doi:10.1371/journal.pcbi.1005641)
Supplement: S2 Text — (PDF) [file pcbi.1005641.s002.pdf]

Supplementary information: SQL queries

ChEMBL (version 21) was downloaded from the database's ftp website and loaded into a local MySQL database. Following SQL queries were used to retrieve the data used in the analyses and to retrieve statistics reported in the dataset description section.

1 Dataset retrieval: basic information about assays and the associated publications

```
SELECT
    a.chembl_id AS assay_chemblid
    , a.description AS assay_description
    , c.pref_name AS species
    , b.chembl_id AS doc_chemblid
    , b.pubmed_id AS doc_pubmed_id
    , b.year AS publication_year
    , b.journal AS publication_journal
FROM
    assays a
    JOIN docs b ON a.doc_id = b.doc_id
    JOIN target_dictionary c ON a.tid = c.tid
WHERE
    a.assay_type = 'F'
    and b.doc_type = 'PUBLICATION'
    and c.pref_name in ('Mus musculus', 'Rattus norvegicus')
```

2 Dataset retrieval: Information about compounds tested in each assay

```
SELECT
    a.chembl_id AS assay_chemblid
```

```
    , e.chembl_id AS compound_chembl_id
    , LCASE(e.pref_name) AS compound_name
    , IF(e.max_phase = 4, 'Yes', 'No') AS approved_drug_flag
    , GROUP_CONCAT(f.level5 separator ';') AS atc_codes
FROM
    assays a
    JOIN docs b ON a.doc_id = b.doc_id
    JOIN target_dictionary c ON a.tid = c.tid
    JOIN activities d ON a.assay_id = d.assay_id
    JOIN molecule_dictionary e ON d.molregno = e.molregno
    LEFT JOIN molecule_atc_classification f ON d.molregno = f.molregno
WHERE
    a.assay_type = 'F'
    AND b.doc_type = 'PUBLICATION'
    AND c.pref_name in ('Mus musculus', 'Rattus norvegicus')
GROUP BY
    a.chembl_id, e.chembl_id, e.pref_name, approved_drug
ORDER BY assay_chemblid
```

3 Dataset retrieval: Information about biological targets of other assays reported in the same publication

```
SELECT
    a1.chembl_id AS in_vivo_assay_chemblid
    , b.chembl_id AS doc_chemblid
    , a2.chembl_id AS other_assay_chemblid
    , a2.assay_type AS other_assay_type
    , c2.pref_name AS other_target
    , c2.target_type AS other_target_type
FROM
    assays a1
    JOIN docs b ON a1.doc_id = b.doc_id
    JOIN target_dictionary c1 ON a1.tid = c1.tid
    JOIN assays a2 ON b.doc_id = a2.doc_id
    JOIN target_dictionary c2 ON a2.tid = c2.tid
WHERE
    a1.assay_type = 'F'
    AND a2.assay_type IN ('B', 'F')
```

```
    AND b.doc_type = 'PUBLICATION'
    AND c1.pref_name IN ('Mus musculus', 'Rattus norvegicus')
    AND c2.pref_name NOT IN ('Mus musculus', 'Rattus norvegicus')
    AND c2.target_type != 'UNCHECKED'
ORDER BY a1.chembl_id
```

4 Dataset statistics: Number of all compounds in ChEMBL

```
SELECT
        COUNT(DISTINCT molregno)
FROM
        activities
```

5 Dataset statistics: Number of all assays extracted from publications

```
SELECT
        COUNT(DISTINCT a.assay_id)
FROM
        assays a
        JOIN docs b ON a.doc_id = b.doc_id
WHERE
        b.doc_type = 'PUBLICATION'
```

6 Dataset statistics: Assay type distribution

```
SELECT
        a.assay_type
        , COUNT(*) as count
FROM
        assays a
        JOIN docs d ON a.doc_id = d.doc_id
WHERE
        a.assay_type != 'None'
        and d.doc_type = 'PUBLICATION'
GROUP BY
        a.assay_type
```

7 Dataset statistics: Assay and target type distribution

```
SELECT
    a.assay_type
    , b.target_type
    , COUNT(*) as count
FROM
    assays a
    JOIN target_dictionary b ON a.tid = b.tid
    JOIN docs d ON a.doc_id = d.doc_id
WHERE
    d.doc_type = 'PUBLICATION'
GROUP BY
    a.assay_type, b.target_type
```

8 Dataset statistics: Animal species used in *in vivo* efficacy assays and their taxonomic classification

```
SELECT
    b.pref_name AS species
    , b.tax_id AS tax_id
    , d.l1 AS level1_classification
    , d.l2 AS level2_classification
    , d.l3 AS level3_classification
    , COUNT(*) as assay_count
FROM
    assays a
    JOIN target_dictionary b ON a.tid = b.tid
    JOIN docs c ON a.doc_id = c.doc_id
    JOIN organism_class d ON b.tax_id = d.tax_id
WHERE
    a.assay_type = 'F'
    AND b.target_type = 'ORGANISM'
    AND c.doc_type = 'PUBLICATION'
    AND d.l1 = 'Eukaryotes'
    AND d.l2 IN ('Amphibia', 'Annelida', 'Arthropoda', 'Aves',
                'Echinodermata', 'Lepidosauria', 'Mammalia',
```

```
                'Mollusca', 'Nematoda', 'Platyhelminthes', 'Teleostei')
    AND pref_name != 'Homo sapiens'
GROUP BY
    b.pref_name, b.tax_id, d.l1, d.l2, d.l3
```
